# Liver Cancer: Current and Future Trends Using Biomaterials

**DOI:** 10.3390/cancers11122026

**Published:** 2019-12-16

**Authors:** Sue Anne Chew, Stefania Moscato, Sachin George, Bahareh Azimi, Serena Danti

**Affiliations:** 1 Department of Health and Biomedical Sciences, University of Texas Rio Grande Valley, One West University Blvd., Brownsville, TX 78520, USA; sachin.george01@utrgv.edu; 2 Department of Clinical and Experimental Medicine, University of Pisa, Via Roma, 55, 56126 Pisa, Italy; stefania.moscato@unipi.it; 3 Italian Institute of Technology, Smart Bio-Interfaces, Viale Rinaldo Piaggio 34, 56025 Pontedera (Pisa), Italy; 4 National Interuniversity Consortium of Materials Science and Technology (INSTM), UdR of Pisa, Via G. Giusti 9, 50121 Firenze, Italy; b.azimi@ing.unipi.it; 5 Department of Civil and Industrial Engineering, University of Pisa, Via Roma, 55, 56126 Pisa, Italy

**Keywords:** biomaterials, hepatocellular carcinoma, 3D models, nanoparticles, microparticles, immunotherapy, scaffolds

## Abstract

Hepatocellular carcinoma (HCC) is the fifth most common type of cancer diagnosed and the second leading cause of death worldwide. Despite advancement in current treatments for HCC, the prognosis for this cancer is still unfavorable. This comprehensive review article focuses on all the current technology that applies biomaterials to treat and study liver cancer, thus showing the versatility of biomaterials to be used as smart tools in this complex pathologic scenario. Specifically, after introducing the liver anatomy and pathology by focusing on the available treatments for HCC, this review summarizes the current biomaterial-based approaches for systemic delivery and implantable tools for locally administrating bioactive factors and provides a comprehensive discussion of the specific therapies and targeting agents to efficiently deliver those factors. This review also highlights the novel application of biomaterials to study HCC, which includes hydrogels and scaffolds to tissue engineer 3D in vitro models representative of the tumor environment. Such models will serve to better understand the tumor biology and investigate new therapies for HCC. Special focus is given to innovative approaches, e.g., combined delivery therapies, and to alternative approaches—e.g., cell capture—as promising future trends in the application of biomaterials to treat HCC.

## 1. Introduction

Biomaterials have recently become a powerful tool for the treatment and study of tumors, including liver cancer. The peculiarity of using biomaterials for cancer relies on the variety and versatility of their properties, which allow size, surface charge and chemistry, shape, morphology, and physiochemical properties, to be tuned according to target applications. Being processable from the nano- to the macro-scale, biomaterials-based technologies can tremendously support the therapies available for liver cancer and the comprehension of this disease by enabling advanced in vitro models. In this review, we provide a comprehensive overview of the biomaterial-based approaches released or under development for liver cancer. Starting from a framework of the liver microenvironment, including normal and neoplastic tissue, and the current treatment methods available for hepatic tumors, this review focuses on highlighting the biomaterials-based therapies that have been developed for liver cancer, which encompass materials used for systemic and local delivery. A discussion of the bioactive factors and targeting agents which have been applied in biomaterial-based therapies is a subject of this review. In the second part, we describe and discuss those biomaterial-based applications that mimic a liver cancer microenvironment to better study hepatic cancer.

This review aims to provide a thorough discussion on the multiscale application of biomaterials for liver cancer. Nano-to-micro range biomaterials are generally used for treatment, whereas, macroscale materials, in the form of tissue engineering scaffolds, are used for study this tumor. There are many review articles on nanotechnology/nanomedicine for liver cancer therapy, such as nanoparticles (NPs) and liposomes [1,2,3,4,5,6,7,8], liver cancer diagnosis and therapy [9], and biomaterials used for targeted systems [10]. However, there are no comprehensive reviews of biomaterial-based therapy that includes an overview of materials in the nano-to-macro domain, which also includes microparticles and scaffolds, with a discussion of both systemic and local delivery applications. To the best of our knowledge, there are no reviews that also discuss the application of biomaterials for 3D systems to better study liver cancer, and in particular hepatocellular carcinoma (HCC). This review article aims at summarizing and discussing the state-of-the-art biomaterials used to treat and study HCC to provide a comprehensive and influential scientific base to support future strategic directions in the therapies of this dismal tumor.

## 2. Liver Structure and Function

**Figure 1:**
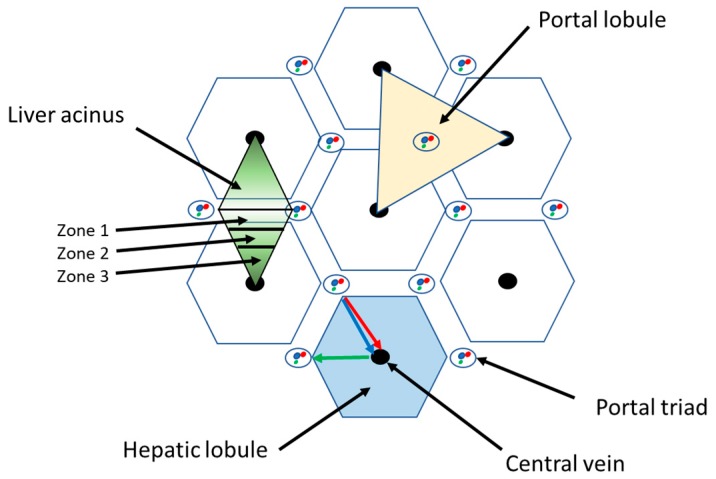
Schematic of the structure of liver lobule.

Liver is a solid organ which is located under the diaphragm in the upper right quadrant of the abdominal cavity. It is closely associated with the small intestine in order to process the venous blood derived from the digestive tract. The liver performs a number metabolic functions, such as bile secretion, bilirubin and nutrient (e.g., fats, proteins, carbohydrates) metabolism, metabolic detoxification, mineral and vitamin storage, as well as some endocrine and immune functions. As a consequence of its key metabolic role, liver pathology, either acute or chronic, can lead to severe outcomes that may progress to death [11]. From an anatomic point of view, human liver is divided into four lobes designated as left, right, caudate, and quadrate. The liver also contains the gallbladder, a sac-like structure for bile accumulation. Every lobe of the liver is divided into lobules by connective tissue branches departing from the connective capsule that covers the liver. These connective septa form the liver stroma, which supports the parenchyma, containing the liver cells, hepatocytes, and act as a scaffold for the blood and lymphatic vessels, and bile ducts. The “hepatic lobule” is the anatomic model used to represent liver lobules [12]. It has a hexagonal-like shape into which hepatocytes are arranged as plates departing from the central vein in the central axis of each lobule. The vertices of the lobules are characterized by the presence of the portal triads: each triad contains a branch of the portal vein and of the hepatic artery and a bile duct (Figure 1).

In the lobule, it is possible to identify the so-called ‘liver acinus’ that is defined as a mass of hepatocytes aligned around the arteriolar and venous blood flow of the portal triads. In this unit, hepatocytes can be divided into three zones, which have different functionalities related to their distance from the oxygen supply. Hepatocytes belonging to the zone 1 are those cells which receive the largest blood supply due to their proximity to the hepatic artery and portal vein branches. Liver cells belonging to zone 2 receive a lesser blood supply, and the hepatocytes in zone 3 are the most sensitive to hypoxia (Figure 1).

Another morphological representation of liver parenchyma is the “portal lobule”, which takes into account the liver biliary excretion function. This structure is represented by a triangle with three adjacent central veins as vertices and a portal triad in the center. Bile is drained from the central veins towards the bile duct of the portal triad (Figure 1).

The liver receives about 80% of the blood from the portal vein, which drains all the blood from intestine, and 20% of the blood from hepatic artery. Blood from these vessels mixes together into sinusoids and flows towards the hepatic vein that brings the blood into the inferior vena cava (IVC) and then to all the body. Hepatocytes represent roughly the 60% of the liver cells; they are often binucleated, and can perform diverse functions, including the metabolism of carbohydrates, lipids and proteins; the production of bile, albumin, and of coagulation factors; as well as the metabolism of steroid hormones and of xenobiotic drugs. Hepatocytes have a sinusoidal face which is directly soaked by the blood flowing into the sinusoids while their lateral faces are in contact to each other and partially modified to form bile canaliculi that convey bile into the bile ducts and finally into the gallbladder. Hepatocyte morphology and function vary depending on their proximity to the periportal or to the pericentral zone as described above [12].

**Figure 2:**
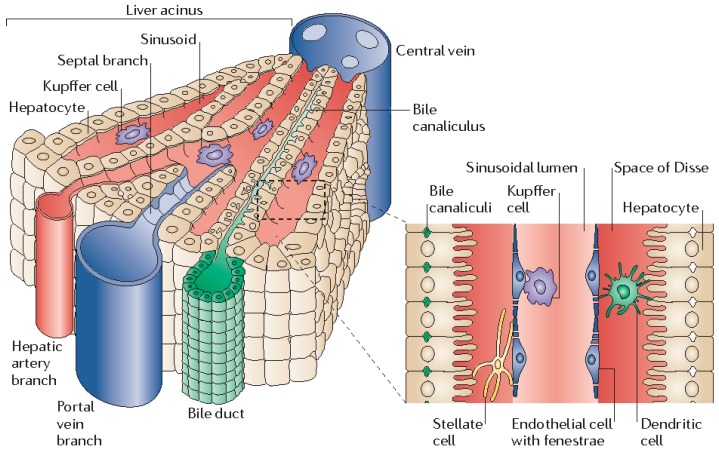
Three-dimensional structure of a liver lobule. Reprinted with permission from Springer Nature Publishing AG, Adams et al., *Nat. Rev. Immunol*., 2006 [15].

Liver also hosts the so-called hepatic progenitor cells (HPCs), known as oval cells (OCs) in rodents, that are considered intra-hepatic precursor cells able to proliferate and differentiate into the two epithelial lineages of the liver—namely, hepatocytes and cholangiocytes—when hepatocyte proliferation is impaired or suppressed [13,14]. Furthermore, in the liver sinusoids, Kupffer cells, which have a phagocytic activity whereas, in the space of Disse (i.e., between the sinusoidal face of the hepatocytes and the sinusoidal endothelial cells), stellate cells (also known as Ito cells) which store vitamin A and are able to regulate inflammatory responses, can be found (Figure 2) [15].

## 3. Liver Neoplasia

Liver tumors are a heterogeneous neoplasia with a great clinical relevance due to morbidity and mortality rates. Hepatic neoplasms can be classified as: (1) primary tumors, when derived from the liver parenchyma such as hepatocellular carcinoma (HCC), intrahepatic cholangiocarcinoma (iCC), fibrolamellar carcinoma and hepatoblastoma (an early liver tumor in children); and (2) secondary tumors, when derived from metastatic cells of other tumors (e.g., colorectal, pancreas, stomach, breast, ovary cancers, and so on) [16].

Among all these neoplasms, HCC is the most common and a constantly increasing incidence in the last decades. HCC has become the fifth most common cancer diagnosed and the second leading cause of death in the world, with prevalent mortality rates in men [16].

The most frequent risk factors for HCC development are chronic infections with hepatitis B virus (HBV) and C virus (HCV), even though in the last years they have become less significant among young people [17]. The vaccination campaigns to prevent HBV, new pharmacological treatments, early diagnosis, and a more diffuse knowledge on the possible transmission routes of these virus, have led to a decrease of chronic viral liver diseases. Nevertheless, other risk factors for HCC have demonstrated a growing incidence, such as diabetes, obesity, alcohol and tobacco consumption, metabolic disease, heritable syndromes, and aflatoxins. Such factors are indeed prevalent in some areas of the world—such as Europe, and North and Latin America—where HCC shows an unfavorable growing trend. Differently, in East Asia, where HCC mainly occurs from viral infections or aflatoxins exposure, a decrease or steady trend has been identified [17].

### 3.1. Diagnosis

**Figure 3:**
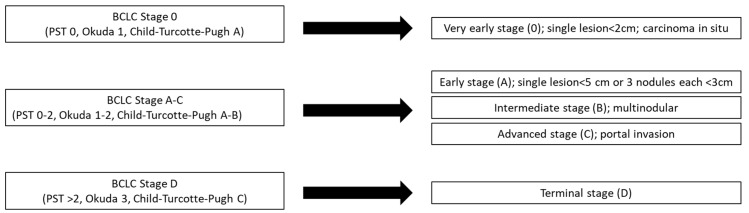
Barcelona-Clinic Liver Cancer (BCLC) criteria.

Thanks to the advanced imaging techniques, surveillance guidelines and follow up programs have been established in different countries for patients with chronic liver disease of any etiologies, to perform tumor diagnosis and staging. Ultrasound is the primary screening method used to evaluate liver integrity and identify hepatic lesions. Other imaging techniques, such as the contrast-enhanced computer tomography (CT) or magnetic resonance imaging (MRI), are employed to improve the diagnosis and describe the staging of the tumor [18]. These radiological methods established criteria which allow liver lesions and staging systems set up for HCC, such as the Barcelona-Clinic Liver Cancer (BCLC) (Figure 3), to be categorized, thus helping the correct diagnostic evaluation [19]. Nevertheless, atypical imaging appearance may require histological analysis.

The immunohistochemical detection of antigens, such as Glypican 3, heat shock protein 70, glutamine synthetase and clathrin heavy chain, is used to confirm the diagnosis for HCC. Biopsy can fail to achieve sufficient sensitivity and specificity due to sampling errors or differential diagnosis misunderstanding (e.g., between early-stage HCC and dysplastic nodules) [20]. Genomic expression profiles have extensively been described for HCC, by means of new biomolecular technologies, namely, microarrays and next-generation sequences (NGS) and correlation between the expression of molecular markers and pathological features, etiology, and clinical outcomes have been proposed [19]. Despite these developments, up to now there are no therapies targeting the major mutations of HCC and no biomarkers able to predict the real response to a specific therapy [21]. To date, protocols of study and evaluation of tumor staging have been drawn up, making it possible to identify the most appropriate therapeutic intervention. BCLC criteria are the most used to establish HCC staging as they consider the tumor characteristics, the performance status of the patient and the liver functional status; finally, they suggest a potential treatment which can increase the patient’s benefit and survival. The staging classification and the treatment schedule can be applied to the majority of HCC patients, although a special consideration has to be warranted to patients with impaired liver function who are candidate for liver transplantation [22]. Based on the staging of HCC, different treatments can be applied to treat this disease.

### 3.2. Current Treatment Methods

#### 3.2.1. Surgical Resection

This treatment has the best performance in patients in very early or early stages of the tumor (BCLC 0-A), without cirrhosis and with variable tumor size, although the tumor extension is a poor prognostic factor [23]. New algorithms for surgical strategies have been proposed to treat HCC patients with BCLC stages B and C by considering the tumor burden, the functionality of the liver reserve and the volume of the remnant liver. There are different scoring systems for estimating liver functional reserve, including Child–Pugh–Turcotte (CPT), the model for end-stage liver disease (MELD), and the aspartate transaminase-to-platelet ratio index (APRI). However, the predictive potential and the results produced by their application are not homogeneous since they do not take into account the patient’s performance status. Recently, the serum bilirubin/serum cholinesterase (BILCHE) scoring system has successfully been applied for selecting patients who can undergo surgical resection with a low risk of postoperative complications [24]. Currently, only a low percentage of well-selected patients can benefit of surgical resection. Recurrences after this approach are unfortunately frequent due to microscopic vascular invasion and tumor dissemination or de novo tumor formation. Adjuvant therapies applied after surgical resection, such as internal radiation therapy or immunotherapy, have been shown to reduce recurrence of tumors, thus prolonging the overall survival [25,26,27].

#### 3.2.2. Liver Transplant

Live transplant is the gold standard treatment in patients with liver cirrhosis and with tumor lesions that subside into Milan criteria (MC) (i.e., patients who have a single tumor mass greater than 5 cm, or up to three lesions of 3 cm each). MC have been proposed by Mazzaferro et al. in 1996 [28], and were adopted globally to select patients for liver transplantation: their application has resulted in a 75% patients with a 5-year overall survival rate and only a 15% patients with tumor recurrence. Although these criteria have been considered too restrictive, it has been observed that tumors exceeding the MC have a higher recurrence and an increased risk to develop different tumor type leading to a poor prognosis against those that fulfill MC. Indeed, many studies proposing expanded criteria still require additional validation (UCSF, UCLA) [23].

#### 3.2.3. Ablation

Ablation procedure is performed by injection of ethanol inside the tumor or by intratumor radiofrequency which produce a necrotic area of about 3 cm diameter. It is a standard of care for patients classified as BCLC A or 0 with tumor size less than 2 cm who cannot undergo or are considered not to benefit from a surgical. Recently, microwave ablation has been applied in patients with larger tumors showing a higher capability of inducing necrosis than radiofrequency. However, these tumor treatments and other emerging ablative methods have shown a great efficacy only in small-size tumors and present the same recurrence rate as observed after surgical resection [23,29].

#### 3.2.4. Chemoembolization

Transarterial chemoembolization (TACE) is a non-surgical procedure that combines embolization with local delivery of drugs; as such, it is preferentially applied in patients with high HCC staging (i.e., BCLC stage B) [29]. This therapeutic treatment has also been applied using drug-eluting beads which can release the chemotherapeutic agent in a controlled manner by concentrating the drug in the tumor site and reducing its systemic circulation. As an alternative to the use of chemotherapeutic agents, the use of radioactive isotopes (i.e., radioembolization; RE) such as Yttrium-90 has recently been proposed. The advantage of RE compared to TACE is that the microspheres used for RE have a smaller size than the particles used for drug-eluting, thus reducing ischemia to the tumor and the live tissue [29]. The best survival benefits from this procedure have been obtained in patients with preserved liver function and without vascular or extrahepatic invasion of the tumor.

#### 3.2.5. Chemotherapy

For patients classified as BCLC D, with advanced progression of HCC and metastasis, systemic chemotherapy is the only treatment that can be applied to try to prolong overall survival. In the latest years, the first line therapy is the chemotherapy drug Sorafenib (Sor) which is a tyrosine kinase inhibitor (TKI). This drug functions by blocking cell surface tyrosine kinase receptors (TKRs) and/or intracellular serine/threonine kinases, thus resulting in anti-angiogenic and anti-proliferative properties. Sor treatment determines an increase of the overall survival and a delay in the progression of the pathology. Despite these benefits, Sor has different severe side effects, such as diarrhea, arterial hypertension, and dermatological adverse events like hand-foot skin reaction (HFSR), which may require to stop the treatment [1]. Due to the limited efficiency of this therapy, other single therapeutic agents have been investigated and are under study in clinical trials. Among different anti-angiogenic TKI investigated in phase III trials, Lenvatinib has been demonstrated to reach a comparable or even better efficacy than Sor. It has been considered as the alternative first line therapy for HCC treatment. Nevertheless, also Lenvatinib can cause many adverse effects as hypertension, diarrhea anorexia, fatigue, and weight loss [30].

In patients who do not tolerate Sor, Regorafenib, a multi-kinase inhibitor of vascular endothelial growth factor receptor 1-3 (VEGFR1-3), has been approved as a second line therapy for HCC, due to the survival benefits observed. In addition to multi-kinase inhibitors, different researches are evaluating molecules able to block the different molecular pathways underlining hepatocarcinogenesis, or agents that target the epigenetic dysregulation described for HCC. Tivantinib, which selectively inhibits c-MET and HGFR, Everolimus, an mTOR inhibitor, or molecules which selectively block VEGFRs, such as Axitinib or Apatinib are a subject of study. It has been observed that a single targeted agent is less efficient than combination of multiple molecules for treating of HCC [31]. Nevertheless, it has been observed that the combination of Sor with either TKI, such as erlotinib [32], or mammalian target of rapamycin (mTOR) inhibitor (i.e., everolimus) [33], did not improve Sor efficacy. Moreover, the combined therapy of Sor with the MAPK/ERK inhibitor Refametinib showed an increase in patient survival but also caused grade 3 and 4 adverse events [34].

Together with the development of new pharmacological agents, many cytotoxic agents as doxorubicin (DOX), cisplastin, 5-fluorouracil, and epirubicin have been also used as systemic therapy for HCC but they did not show high improvements in overall survival and their combination caused different side effects like an increase of myelotoxicity or neurotoxicity, neutropenia compared to the single agent administration. Furthermore, different studies have investigated the combination of therapies of Sor with TACE or with these systemic agents. They reported that the administration of Sor in combination with one or more inhibitors may improve the benefits for the patient, although toxicity and adverse effects may increase accordingly [31].

#### 3.2.6. Immunotherapy

Immune therapy has recently been introduced to treat HCC. The immune-based approach relies on the observation that tumor cells can survive by escaping the immune system when they express, on their surface membrane, the programmed cell death ligand 1 (PDL-1), which is able to bind the programmed death-1 (PD-1) receptor on activated T cells. The interaction between these ligands leads to the inactivation of T cells and consequently to the tumor cells survival [21]. Nivolumab is a human monoclonal antibody currently used for different metastatic tumors: it has the ability to bind to PD-1 receptor interfering with the PDL-1 interaction and thus restoring the immunocompetence of T cells against cancer. Similarly, Tremelimumab is used to block the cytotoxic T-lymphocyte antigen (CTLA-4), allowing T cells to continue tumor cell destruction [35]. Clinical trials are ongoing to evaluate the efficiency of these immune therapies as alternative treatments for HCC and point out the possible adverse effects of their application either as single therapies or in combination with TACE or other therapeutic approaches.

Recently, it has been observed that immunotherapy induced adverse effects such as rush, fatigue, pruritis, and diarrhea and the increase of aspartate aminotransferase/alanine aminotransferase worsening liver functionality. Cardiovascular toxicities have been also observed during the treatment with immune checkpoint inhibitors [36]. Furthermore, in some cases, immune cell infiltration and a delayed antitumoral response was demonstrated leading to the so called pseudoprogression of the tumor [37].

Another therapeutic approach under evaluation for HCC treatment is based on the use of viruses able to induce tumor cell death by activating the immune system. Second generation oncolytic human herpes simplex virus type-1 has been genetically engineered to express the granulocyte macrophage colony stimulating factor. It has been observed that oncolytic viruses are efficient against different tumors blocking their growth and progression [38]. The induction of anti-tumor immunity using tumor-associated antigens (TAAs) as peptide vaccines is a key research field, also applied to HCC [39]. For example, glypican protein 3 (GPC3) has been used as a potential peptide vaccine for its ability to induce the cytotoxic T lymphocyte (CTL) response [40]. Furthermore, GPC3-derived epitope peptide conjugated to liposomes seemed to be able to induce CTLs to inhibit the growth of GPC3-expressing tumor [41]. Immune targeting of multi-drug resistance protein 3 (MRP3) was suggested to induce CTL response against HCC cells which overexpress this transporter [42] and C-35 peptide from HCV-core protein was shown to induce a specific IgG1 response [43].

#### 3.2.7. Gene Therapy

MicroRNA (miRNA) and small-interfering RNA (siRNA) have been studied as nucleic acid-based therapeutic drugs: miRNAs are non-coding single-strand molecules which act at post-translational level or silencing RNAs [44]; siRNAs are double-stranded RNAs with the capability of specifically targeting for degradation at several sites of antisense strand mRNA, thus knocking down the expression of a specific gene [45]. The application of miRNAs and siRNAs is limited by their off-target effects that can induce silencing of healthy genes. Moreover, delivery of these nucleic acids has many obstacles such as nuclease degradation and body filtration [46]. In HCC, as in many tumors, miRNA profile is altered compared to that of healthy subjects, showing both upregulation and downregulation of miRNAs [47]. Some of these miRNAs are considered new possible biomarkers (e.g., miRNA-21), while some others are potent tumor suppressors, like miRNA-34a, miRNA-29, and miRNA-122, as they are downregulated in HCC patients [48,49]. As demonstrated by Deng and coauthors in 2011, their restoration can inhibit tumorigenesis using nanoparticles loaded with Rubone, a natural compound able to upregulate miRNA-34a [50].

## 4. Biomaterials for Drug Delivery in Liver Cancer

Cancer including HCC is a complex and diverse disease, which presents many challenges to diagnose and treat it. The versatility and complex nature of biomaterials presents a promising approach to address some of these challenges. Such biocompatible materials can be easily tailored and engineered to be utilized to study, diagnose, and treat liver cancer by altering their different properties. Hereafter, we discuss the different biomaterials that have been developed for improving drug delivery in HCC applications, which are summarized in Table A1 (Appendix A).

### 4.1. Particles

#### 4.1.1. Nanodiamonds

**Figure 4:**
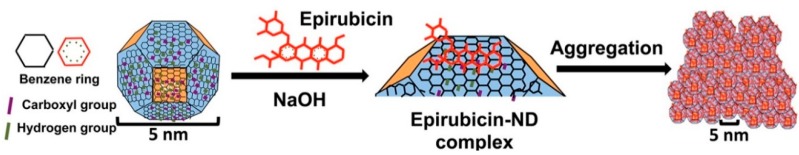
Schematic model showing surface and chemical structure of nanodiamond (ND) and Epirubicin (Epi) and the synthesis and aggregation of nanodiamond–Epirubicin drug complex (EPND). Reprinted with permission from ACS Publications, Wang et al., *ACS Nano*, 2014 [53].

Nanodiamonds are approximately 5 nm in diameter and are made out of truncated semiotahderal carbon structures [51]. By varying the surface electrical potential of the material by altering the functional chemical groups on their surfaces, nanodiamonds can be used to load a wide range of bioactive therapeutic factors for cancer therapy [52]. Nanodiamonds are more biocompatible and well tolerated than other nanocarbon materials [53]. Wang et al. used nanodiamonds to deliver epirubicin, a chemotherapeutic drug to chemo-resistant cancer stem cells [53]. As seen in Figure 4, the drug was physically adsorbed onto the nanodiamonds. The nanodiamond-epirubicin particles resulted in a nanodiamond aggregate diameter of 89.2 ± 3.3 nm.

The epirubicin delivery with nanodiamonds resulted in enhanced endocytic uptake and tumor cell retention compared to the drug delivered alone, and thus enhanced the killing of cancer stem cells and non-cancer stem cells. The release from the nanodiamonds can be stimulated intracellularly by charged proteins and thus ensuring intracellular-specific drug release which results in decrease in toxic side-effects that can result from systemic delivery. These nanodiamonds are able to overcome chemoresistance by prevention of the efflux of the drug by ABC transporters. Wang et al. demonstrated that optimized nanodiamonds can efficiently load the drug, facilitate passive transport to cancer cells and intratumoral release of the drug making it a promising candidate for hepatic cancer treatment.

#### 4.1.2. PLGA-Based NPs

**Figure 5:**
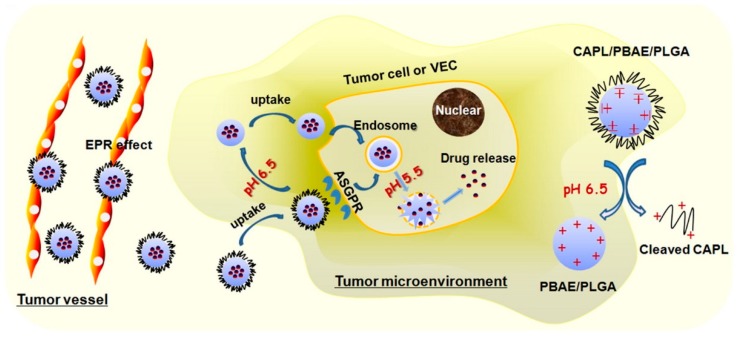
Schematic of hepatoma-targeting and stepwise pH-responsive mechanisms of CAPL/PBAE/PLGA NPs. Reprinted with permission from Elsevier, Zhang et al., *Journal of Controlled Release*, 2016 [57].

The copolymer poly(lactic-co-glycolic acid) (PLGA) is a polyester that is often used as a biomaterial for the delivery of bioactive factors for different applications including cancer, due to its great biocompatibility, biodegradability, and versatility [54,55]. Furthermore, the degradation rate of PLGA can be optimized by altering the lactic to glycolic acid ratio, molecular weight and the end cap groups, the latter also providing different options for drug affinity. PLGA has been used extensively for drug delivery, including applications for the treatment of HCC. Ma et al. used PLGA NPs to deliver 5-fluoruracil for the treatment and imaging of HCC [56]. Besides using this copolymer by itself, many groups have also used PLGA in combination with other polymers. Zhang et al. designed a stepwise pH-responsive NP system containing charge reversible pullulan-based (CAPL) shell and poly(β-amino ester) (PBAE)/PLGA core to deliver paclitaxel (PTX) and combretastatin A4 (CA4), as a combination antiangiogenesis and chemotherapy to treat HCC (Figure 5) [57].

**Figure 6:**
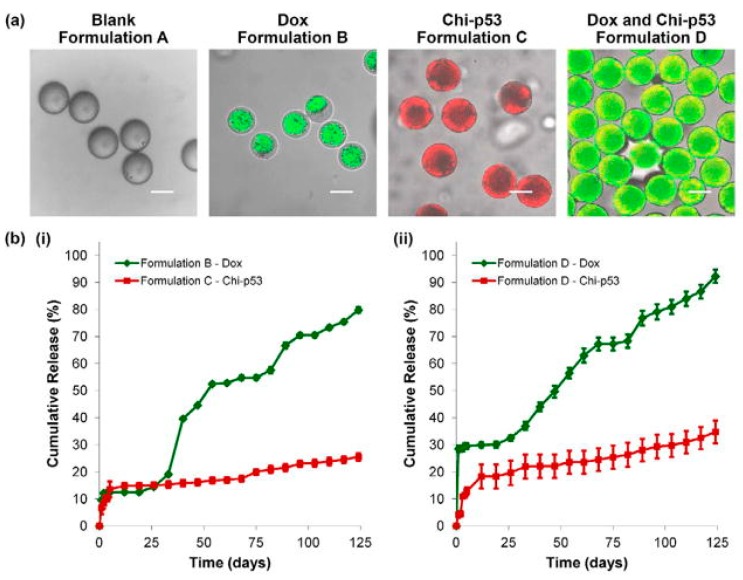
(**a**) Transmitted light and laser scanning confocal (overlay) micrographs of blank and drug loaded double-walled PLLA (PLGA) microspheres. The distribution of DOX in Formulations B and D microspheres is indicated in green. The distribution of chi-p53 NPs in formulations C and D microspheres is indicated in red and yellow (colocalization of red and green), respectively. Scale bar = 50 μm. (**b**) In vitro DOX and chi-p53 release from double-walled PLLA(PLGA) microspheres. Reprinted with permission from Elsevier, Xu et al., Biomaterials, 2013 [58].

The NPs respond to: (*i*) the cleavage of β-carboxylic amide bond in CAPL in weakly acidic tumor microenvironment (pH ≈ 6.5), and (*ii*) the ‘proton-sponge’ effect of PBAE in endo/lysosome (pH ≈ 5.5), thus releasing CA4 and PTX in a two-step process. Zhang et al. demonstrated that the CAPL/PBAE/PLGA NPs exerted increased inhibitory effects of PTX and CA4 on tumor growth and angiogenesis. Xu et al. designed double-walled microspheres that consisted of a poly(D,L-lactic-co-glycolic acid) (P_DL_LGA) core surrounded by a poly(L-lactic acid) (PLLA) shell layer [58]. The particles were loaded with the chemotherapeutic drug DOX in the core and chitosan-DNA NPs containing the gene encoding the p53 tumor suppressor protein (chi-p53) in the shell (Figure 6). The combined treatment with DOX and chi-p53 demonstrated enhanced cytotoxicity as compared to either DOX or chi-p53 treatments alone. The double-walled particles were prepared using a where precision particle fabrication (PPF) apparatus equipped with a coaxial nozzle that produce a jet of core P_DL_LGA surrounded by an annular stream of PLLA and allowed the simultaneous release of the two bioactive factors.

Liang et al. used NPs synthesized with block copolymers poly(*γ*-glutamic acid) (PGA) and poly(lactide) (PLA) to deliver PTX [59]. PGA is often incorporated as a biomaterial since it is water soluble, biodegradable, non-toxic, and naturally occurring. The *γ*-PGA was conjugated with the active PLA to create the block polymer [59]. It was then loaded and conjugated again with the galactosamine as the targeting agent. These targeted NPs enhanced the tumor reduction efficacy in comparison to non-targeting NPs and previously established/clinically available forms of PTX. PLGA has also been used in combination with poly(ethylene glycol) (PEG), which is widely applied in many biomedical applications due to its biocompatibility and high hydrophilic properties. Ni et al. developed biotin-/lactobionic acid–modified poly(ethylene glycol)-poly(lactic-co-glycolic acid)-poly(ethylene glycol) (BLPP) copolymer NPs to deliver curcumin- and 5-fluorouracil [60]. The biotin and lactobionic acid served as dual targeting agents and they were linked to the amphiphilic triblock PEG-PLGA-PEG. Wang et al. used polyethylene glycol-*block*-polylactic acid (PEG-PLA) amphiphilic copolymer assembled in water with oligo or polylactide-drug conjugates to deliver SN38 (7-ethyl-10-hydroxycamptothecin) [61]. Owing to its low solubility in water and dose-limiting toxicity, SN38 use in the clinic is hindered. Wang et al. used these small-sized and ultrastable nanomedicines to deliver SN38 as better tolerated cytotoxic nanotherapy. The covalent conjugating of the drug augmented its retention in the PEG-PLA NPs. By increasing the length of the PLA chain, the retention and antitumor activity was increased, enabling a complete eradication of the tumors in mice.

PLGA NPs have been widely used for cancer therapy; however, groups are moving towards using a combination of PLGA with other synthetic or natural polymers to create optimal drug delivery vehicles by leveraging the benefits of the different polymers. PLGA is very versatile in its ability to adjust drug release rates; on the other hand, other polymers may be able to more efficiently load the drugs of interest. For example, PBAE which has piperidine rings that can better carry the PTX and CA4 drugs investigated by Zhang et al. [57]. However, PBAE alone is not a suitable polymer for drug delivery, as it has poor stability in blood and has high positive charges [62]. Thus, combination with PLGA, as shown by Zhang et al., resulted in NPs with enhance stability and adjustable release kinetics due to the PLGA and better loading bioavailability due to the PBAE [57]. 

#### 4.1.3. Natural Polymers Particles

**Figure 7:**
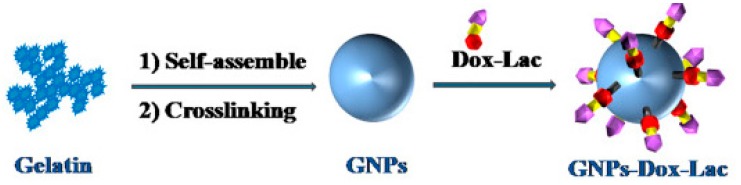
Preparation of GNPs-DOX-Lac particles. Reprinted with permission from Elsevier, Liu et al., *Nanomedicine: Nanotechnology, Biology and Medicine*, 2018, [65].

Natural polymer, unlike synthetic polymers, are naturally available in the environment. Gelatin is a widely used and biocompatible protein, obtained as denatured collagen, that can be degraded by metal matrix protease-2 (MMP2), which is over-expressed in tumors [63,64]. Liu et al. used gelatin NPs to deliver DOX-lactose [65]. The NPs were constructed by self-assembly followed by crosslinking of the gelatin (Figure 7). In fact, unlike collagen, non-crosslinked gelatin is soluble at 37 °C.

DOX-lactose molecules were then conjugated to the particles where lactose was utilized to target HCC cells. The tumor extracellular matrix MMP2 leads to the degradation of the NPs and release of DOX. In addition, there was a pH-responsive disassociation of DOX and lactose in the tumor cells, resulting in free drug inside the cells. This method reduced the systemic toxicity generally produced by DOX while increasing tumor inhibition rates. Hanes et al. used gelatin and chondroitin-6-sulfate microspheres to deliver interleukin-2 (IL-2) for immunotherapy [66]. This microsphere was created using an aqueous-based complex coacervation process. Through the use of microsphere mediated IL-2 release, prolonged liver-specific IL-2 exposure was observed. Hanes et al. demonstrated that the microspheres were more effective than an optimized dose of autologous cells, which are currently considered the gold standard for immunotherapy.

Chitosan is a cationic polysaccharide which is manufactured commercially through partial deacetylation of chitin [67]. Chitosan NPs (CNPs) are usually fabricated through cationic chitosan ionotropic gelation with polyanion sodium tripolyphosphate. Qi et al. demonstrated that CNPs not loaded with any drug have antitumor effect on HCC cells due to neutralization of cell surface charge, decrease of mitochondrial membrane potential, and induction of lipid peroxidation [68]. CNPs cause death by disrupting the cell membrane, decreasing the negative surface charge and mitochondrial membrane potential, inducing lipid peroxidation, disturbing the fatty acid composition of the membrane and fragmenting DNA. Xu et al. further showed that CNPs inhibit tumor growth through tumor angiogenesis impairment [67]. The vascular endothelial growth factor receptor 2 (VEGFR2) levels was decreased, resulting in blockage of VEGF-induced endothelial cell activation. CNPs have also been used to deliver other molecules and drugs. For example, Bu et al. utilized these particles to delivery trans-resveratrol [69], and Cheng et al. [70] utilized these particles for gene therapy. Chitosan ability to induce cancer cell death by itself and act also as a drug delivery vehicle make it is a very promising natural polymer for hepatic cancer treatment.

#### 4.1.4. Metallic NPs

Metallic NPs such as iron, gold and silver have been used in different biomedical applications for imaging and therapy. Magnetic iron NPs have the ability to be controlled by superparamagnetism, allowing the regulation of their movement and concentration using an external magnetic field [71]. Xue et al. utilized galactosylated-carboxymethyl chitosan-magnetic iron oxide NPs (Gal-CMCS-Fe_3_O_4_-NPs) to deliver the tumor suppressor gene RASSF1A [72]. They were able to improve the transfection efficiency of the particles by controlling the direction of travel of the super paramagnetic iron oxide NPs. Kou et al. focused on ultrasmall superparamagnetic iron oxide (USPIO) NPs conjugated with humanized SM5-1 antibody, which binds to HCC cells [73]. These particles can be used as contrast agents for diagnosis by MRI. Gold NPs main accumulation site is in the liver, and thus, it is a promising biomaterials for the treatment of liver cancer [74,75]. Gold NPs are usually capped with either sodium citrate, polyamidoamine dendrimers (PAMAM), or L-aspartate to stabilize the particles and their cellular uptake is usually mediated by clathrin dependent endocytosis. One advantage of these microparticles are their anti-angiogenic properties by inhibiting endothelial and fibroblast cell proliferation [76]. Tomuleasa et al. reported on gold NPs conjugated non-covalently with L-aspartate and conventional chemotherapy drugs, cisplatin/DOX/capecitabin, to deliver this anti-tumor therapy through endocytic uptake into the cells [77]. Ma et al. used gold NPs conjugated with the antibody for SM5-1 which was stabilized using sodium nitrate [78]. Paino et al. [74] also studied gold NPs which were capped with either sodium citrate or polyamidoamine dendrimers (PAMAM). Finally, Xue et al. used gold NPs to deliver miRNA-375 [75]. Compared to other types of NPs, metallic NPs are promising for the treatment of hepatic cancer due to their ability to be controlled by an electromagnetic field, their broad optical properties which make results in their ability to not only serve as a delivery vehicle, but also as contrast agent for imaging.

#### 4.1.5. Lipid-Based Particles

Liposomes are characterized by a lipid bilayer that is formed from self-assembly of amphipathic phospholipids, resulting in an interior aqueous space. Gao et al. used DOTAP/Cholesterol liposome conjugated with anti-EGFR to target HCC cells. These liposomes were used to deliver adriamycin and ribonucleotide reductase M2 (RRM2) siRNA. Their results demonstrated that the targeting and dual delivery of both drugs gave rise to enhanced therapeutic effects compared to non-targeted or single drug liposomes. Wei et al. investigated liposomes made of soybean phosphatidylcholine/cholesterol which was PEGylated to deliver DOX [79]. PEGylation is advantageous as it increases circulation time and avoids rapid clearance by the reticuloendothelial system [80,81]. Lo et al. also applied PEGylated liposomes to deliver DOX, but the exact construct is unknown [82]. Wang et al. used immunoliposomes to delivery DOX [83]. They used commercially available Ls-DOX, but modified it by adding 1,2-distearoyl-sn-glycero-3-phosphoethanolamine-N-[maleimide(polyethylene glycol)-2000] (DSPE-PEG-Mal) conjugated with Metuximab. Wang et al. also used liposomes, but the construct was not specified. The liposome was loaded with a triple fusion plasmid of monomeric red fluorescence protein, renilla luciferase, and herpes simplex virus truncated thymidine kinase or DOX. In addition, an anti-CD44 antibody was conjugated to the loaded liposome [84]. Unlike liposomes which have lipid bilayer, micelles are a lipid monolayers. Abdelmoneem et al. utilized dual-functionalized spray-dried casein micelles (CAS-MCs) for combined delivery of two phytochemicals; berberine (BRB) and diosmin [85]. Bogorad et al. studied lipidoids, which are lipid-like molecules that are synthesized from alkyl-amines and alkyl-acrylates to deliver siRNAs that knockdown all integrin subunits in hepatocytes [86]. Besides liposomes and micelles, groups have also applied lipid NPs to deliver bioactive factors. As an example, Xu et al. utilized targeted solid lipid NPs to deliver docetaxel. The primary components of this NP were dioleoylphosphatidyl ethanolamine (DOPE) and egg yolk phosphatidylcholine (ePC), which are both biodegradable and biocompatible [87]. Huang et al. also investigated lipid NPs; however, their NPs consisted of 1,2-distearoyl-sn-glycero-3-phosphatidylcholine (DSPC), 1,2-dimyristoyl-sn-glycero-3-phosphoethanolamine-*N*-[methoxy (polyethylene glycol)-2000 (DMPE-PEG2000), cholesterol, and a cationic lipid (RL01) and they were aimed to deliver anti-miR-17. Lipid-based NP ability to easily and spontaneously self-assemble in aqueous environments, from chemical components/monomers that are designed optimally, into vast three-dimensional particles make it promising vehicles for drug delivery.

#### 4.1.6. Other Particles

There are many other types of particles that have been developed using diverse biomaterials to be applied in the treatment of HCC. Among those, Wang et al. used hydroxyapatite NPs to delivery selenium [88]. Hydroxyapatite material itself is able to induce cell death and, thus these NPs act as both a drug delivery vehicle and anticancer agent for HCC [89,90]. Pullulan-based NPs were investigated by both Zhang et al. [57] and Wang et al. [91]. Pullulan is a polysaccharide enabling advantages as it exhibited pH-sensitive properties, thus allowing control release property. There are several other materials that have been used for liver cancer therapy including lipid-coated calcium carbonate NPs [92], galactosamine-conjugated albumin [93], polyisohexylcyanoacrylate [94], dendrimer NPs [95], insulin multimethacrylate particles [96], and glass microspheres [97].

### 4.2. Local Delivery

Compared to systemic delivery, local delivery of therapeutics allows for the decrease of systemic toxicity and increase bioavailability of the drug. By delivering drugs locally, the drug concentration at the tumor environment can be maximize and non-target systemic exposure and organ toxicity can be minimized. Furthermore, local delivery can increase the efficacy of the drug by bypassing the harsh environment and longer journey that the drug has to take to reach the targeted site of interest.

### 4.3. Local Implantation

**Figure 8:**
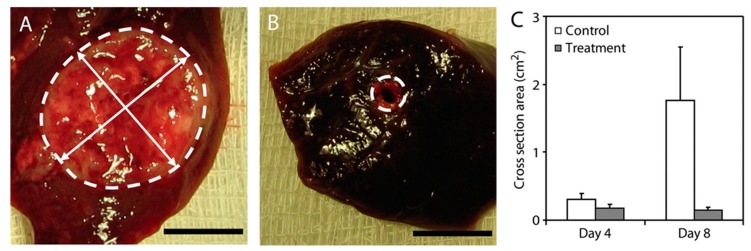
DOX-containing millirods. Photographs a untreated control (**A**) and a treated (**B**) tumor cross section on day 8. The boundary between the tumor and normal liver tissue is indicated with a white dotted outline. The mean cross sectional area of the untreated control and tumors after 4 and 8 days (**C**). The error bars indicate the standard deviation of each measurement (*n* = 4). Reprinted and adapted with permission from Wiley, Weinberg et al., *Journal of Biomedical Materials Research Part A*, 2007 [99].

Che et al. used PCL electrospun nanofibers to deliver both a drug (i.e., PTX) and gene (i.e., miRNA-145) [98]. They showed that the implantation of these biomaterials in the liver after tumor removal significantly decreased the recurrence and improve the overall survival in patients with HCC. Radiotherapy can be often used as adjuvant therapy for the prevention of the recurrence of cancer and/or metastasis after the surgical resection of the tumor. Wang et al. implanted non-degradable glass microparticles to deliver phosphorus-32 for preventive regional radiotherapy after hepatectomy [97]. An absorbable gelatin sponge soaked with the microparticle suspension was buried within the resection surface. This implant was able to significantly decrease the recurrence of HCC after tumor resection. There are several groups that have looked at developing ‘polymer millirods’ that can be implanted in the tumor. Weinberg et al. [99,100] and Qian et al. [101] developed DOX-containing P_DL_LGA cylindrical polymer millirods (length 8 mm, diameter 1.5 mm). Thus, Weinberg et al. demonstrated that these DOX-containing implants were able to provide high DOX concentration at the tumor site, resulting in significant decrease in tumor size compared to the untreated control (Figure 8) [99]. These PLGA millirods have also been used to deliver other chemotherapy drugs such as carboplatin [102] and 5-fluorouracil [103].

Hanes et al. injected gelatin and chondroitin sulfate microspheres loaded with IL-2 near the tumor site. Microsphere degradation was mediated by tumor expression of degradative enzymes like collagenase, leading to the local delivery of interleukin-2 [104]. HCC receives a majority of their blood supply from the hepatic artery compared to the portal vein for normal liver. In TACE, a catheter is placed in the branches of the hepatic artery and chemotherapy agents can be infused into the artery [105]. Drug eluting beads (DEB) is often used in combination with TACE to deliver chemotherapy into adjacent tissue [106,107]. These DEBs are usually made of polyvinyl alcohol (PVA) hydrogel that has been sulphonated to enable the binding of chemotherapy drugs. Hong et al. utilized sulfonate-modified PVA hydrogel DEBs to delivery DOX which was administered intra-arterially in the same exact manner as with TACE [108]. Pawlik et al. applied DOX-DEBs and sorafenib in a Phase II trial [109]. They demonstrated that these combination in unresectable HCC patients were well tolerated and toxicity can be managed with Sor dose adjustment. Besides DEB, microparticles made of PLGA have also been used in combination with TACE. Qian et al. used TACE with mitomycin C-loaded PLGA microparticles [110].

## 5. Biomaterials for Bioactive Factor Delivery in Liver Cancer Therapy and Imaging

### 5.1. Chemotherapy

DOX, also known by one of its trade name Adriamycin, is one of the most common drugs used in liver cancer therapy and in many biomaterial based therapies [58,65,77,79,82,83,93,96,108,109,111]. Besides DOX, taxel-based drugs, such as docetaxel and PTX, are often used in biomaterial based therapies for HCC as well [57,59,87,98]. Liang et al. studied PTX loaded poly(*γ*-glutamic acid)-poly(lactide) NPs to deliver chemotherapy for HCC [59]. PTX is primarily utilized for breast and ovarian cancers, however, as shown by Liang et al., it can also effective in treating HCC [112,113]. Zhang et al. also used PTX, but in combination with combretastatin A4, which will be discussed later in this review. Although it is structurally similar, docetaxel has shown to be more effective than PTX [114]. Docetaxel functions as an inhibitor of the depolymerization of microtubules. Xu et al. loaded docetaxel into the solid lipid NPs [58]. 5-Fluorouracil (5-FU) is a chemotherapeutic drug that disrupts nucleoside metabolism resulting in DNA and RNA disorders [115,116]. Ma et al. utilized this drug by conjugating it to PLA NPs [56]. Tomuleasa et al. tested multiple different chemotherapeutic drugs by loading gold NPs with cisplatin, capecitabine, and DOX.

### 5.2. Radiotherapy

Radiotherapy can be often used as adjuvant therapy for the prevention of the recurrence of cancer and/or metastases after the surgical resection of the tumor. Wang et al. investigated non-degradable glass microparticles to deliver phosphorus-32 for preventive regional radiotherapy [97]. As it decays, this radioisotope emits high energy beta particles. Wang et al. demonstrated that the glass microspheres implanted in the liver was able to significantly decrease recurrence after surgery.

### 5.3. Immunotherapy

Immunotherapy is a promising approach for cancer treatment as it has the ability to develop long-term immunity to the tumor originally treated [117]. Microparticle systems have shown ability to improve immune response. As an example, Hanes et al. used gelatin and chondroitin-6-sulfate microspheres to deliver IL-2 for immunotherapy [66]. Previously, IL-2 has been used to treat melanomas and renal cell carcinoma through systemic means, but it has shown to be toxic in high doses [118]. Genetically-engineered autologous cells are currently the gold standard for immunotherapy [118]. These particles developed by Hanes et al. are relatively easier to manufacture compared to these cells, and thus present a promising approach for cancer immunotherapy. Ma et al. used SM5-1, a monoclonal antibody, as immunotherapy because it binds to p230, a membrane protein overexpressed in HCC [78]. This antibody was shown to impede tumor growth, induce apoptosis, and downregulate tumor angiogenesis [73,119,120,121].

### 5.4. Natural Occurring Compounds

Natural occurring compounds are drugs that can be extracted from plants and animals. Abdelmoneed delivered two phytochemicals extracted from plants; berberine (BRB) and diosmin (DSN) using micelles [85]. Ni et al. delivered both curcumin- and 5-fluorouracil-loaded with biotin-/lactobionic acid–modified PEG-PLGA-PEG (BLPP) copolymer NPs and saw synergistic anticancer effects (i.e., cytotoxicity) in vivo [60]. Curcumin is a polyphenol extracted from turmeric inducing anti-angiogenic, antitumor, and anti-inflammatory physiological responses [122]. Bu et al. also focused on a naturally occurring compound. They loaded trans-resveratrol (as a model drug), a natural plant compound, into CNPs, demonstrating that NPs were more effective on cancer cells compared to trans-resveratrol solution [69].

### 5.5. Gene Therapy

Gene therapy is used to treat a disease by transferring a therapeutic nucleic acid (i.e., plasmid DNA, siRNA, or microRNA) to introduce a new gene or restore, increase or halt gene expression of a gene.

#### 5.5.1. Plasmid DNA

Plasmid DNAs are double-stranded, closed, and circular vectors often used for gene delivery due to recombination techniques that allow genes to be inserted into the plasmid. Xue et al. utilized galactosylated-carboxymethyl chitosan-magnetic iron oxide (Gal-CMCS-Fe_3_O_4_) to deliver the tumor suppressor gene Ras Association Domain Family 1A (RASSF1A) [72], which plays a vital role in apoptosis, cell-cycle regulation, and microtubule stability [123,124]. HCC are lacking RASSF1A expression [125] and thus, the delivery of this plasmid in combination with intraperitoneal injection of mitomycin and an external magnetic field resulted in smallest tumors compared to other groups tested. The re-expression of the RASSF1A gene resulted in increased sensitivity of HCC cells to the chemotherapy drug mitomycin [72]. Xu et al. also combined chemotherapy with gene delivery. They designed a double layered microparticles loaded with the chemotherapy drug DOX in the core and chitosan-DNA NPs containing the gene encoding the p53 tumor suppressor protein (chi-p53) in the shell. The combined treatment with DOX and chi-p53 treatment demonstrated enhanced cytotoxicity as compared to either DOX or chi-p53 treatments alone. The p53 gene is a gene that is often mutated in HCC, mostly in the apoptosis pathway mediated by p53 [126]. Thus, p53 expression using plasmid DNA could help fix the control of normal cell growth.

#### 5.5.2. siRNAs

Small interfering RNAs (siRNAs) are noncoding sequences used to disrupt the effects of mRNAs. Gao et al. combined the delivery of a chemotherapy agent, Adriamycin, and ribonucleotide reductase M2 (RRM2) siRNA with liposome-polycation-DNA complex (LPD) conjugated with anti-EGFR (epidermal growth factor receptor) [127]. RRM2 expression is higher in HCC than in non-HCC tissue and thus RRM2 siRNA is a promising therapy for HCC. Adriamycin may induce upregulation of RRM2 and thus, the combination of Adriamycin and RRM2 siRNA can result in combined therapeutic effects. The combined therapy resulted in enhanced therapeutic effects (i.e., cytotoxicity, apoptosis, and senescence-inducing activity) compared with single-drug loaded or non-targeted controls. Bogorad et al. delivered siRNAs targeting all integrin subunits in hepatocytes [86]. Integrins play an important role during development, regulating cell differentiation, proliferation, and survival. The knockdown of the integrin receptors significantly retarded the progression of HCC.

#### 5.5.3. miRNAs

MiRNAs are also noncoding RNAs that are around 22 nucleotide long that modulate mRNA stability and expression. MiRNAs can target multiple mRNAs simultaneously, while siRNAs can target only one at a time [128]. Zhou et al. studied the delivery of the *let-7g* tumor suppressor miRNA using dendrimer NPs that mediate miRNA delivery to late-stage liver tumors with low hepatotoxicity [95]. Huang et al. delivered antimiR-17 with a lipid NP. MiR-17 is an oncogene, and ectopic expression with miR-17-3p results in the development of HCC in a mouse model [129,130]. The suppression of this oncogene through antimiR-17 delivery hindered the proliferation of HCC cells. Although a first-line drug for HCC, Sor often induces resistance due to autophagy [92]. Thus, Zhao et al. investigated the delivery of Sor in combination with miR-375, an inhibitor of autophagy with lipid-coated calcium carbonate NPs. Xue et al. also delivered miR-375, but using gold NPs [75]. Finally, Che et al. delivered miRNA-145 in combination with PTX from an electrospun PCL scaffold [123].

### 5.6. Imaging

**Figure 9:**
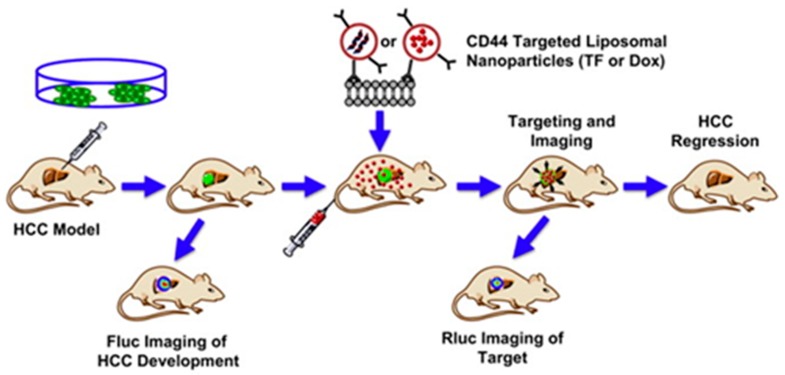
Schematic of targeted liposomes for imaging and therapy of HCC. The HCC model was developed by in situ injection of DF (Fluc, GFP) HepG2 cells with the progression or regression of HCC bearing tracked by Fluc imaging in vivo. The targeting of CD44 conjugated liposomes can be tracked by Rluc imaging. HCC regression resulted from administration of GCV and DOX. Reprinted with permission from Elsevier, Wang et al., *Biomaterials*, 2012 [84].

Imaging in cancer therapy is vital to assess tumor growth and/or ensure and track the delivery of treatments. Wang et al. applied liposomes to deliver a reporter system imaging agent which allowed them to monitor and evaluate the efficiency of targeting the tumor [84]. The triple fusion (TF) plasmid, consist of monomeric red fluorescence protein (RFP), renilla luciferase (Rluc), and herpes simplex virus truncated thymidine kinase (HSV-ttk) reporter genes and was used to monitor the response of the tumor to DOX delivered using the same liposome system. Bio-optical bioluminescence imaging (BLI) was used to monitor tumor growth status by Fluc and targeting of the liposomal particles to the site by Rluc (Figure 9).

Besides being used as drug delivery vehicles, ultrasmall superparamagnetic iron oxide can be employed as contract agent in MRI. Indeed, Kou et al. utilized these particles for diagnostic imaging of HCC [73].

## 6. Targeted Delivery

Systemic delivery systems often result in the accumulation of drugs in the mononuclear phagocytic system cells in the liver, spleen, and bone marrow [131], and increase in hematologic toxicities due to circulation time [132]. Targeting agents to tumor cell surface antigens or receptors allow biomaterials to effectively and efficiently deliver bioactive factors to the cells of interest while decreasing side effects to non-targeted cells.

### 6.1. Antibodies

Antibodies have evolved into tumor targeting agents for drug delivery. Gao et al. used NPs conjugated with anti-epidermal growth factor receptor (EGFR) since HCC cells overexpress EGFR [127]. They found that the NPs can be efficiently delivered to EGFR-overexpressing or moderately-expressing HCC cells, but not to the HCC cells with low levels of EGFR. Wang et al. selected anti-CD147 antibodies to target HCC cells [83]. CD147 is an important marker in the progression of HCC and thus, it is also highly expressed on the surface of HCC cells. Due to the EPR effect, the liposomes aggregated on the tumor and then absorbed through ligand mediated internalization. Wang et al. used anti-CD44 as a targeting agent to target cancer stem cells (CSCs) for HCC therapies [84]. Ma et al. employed SM5-1, a humanized mouse antibody that has a high binding affinity for a membrane protein, p230, a 230 kDA membrane protein overexpressed in HCC cells, in PLA NPs [56]. Ma et al. also used gold NPs conjugated with SM5-1. This antibody was also used by Kou et al. with ultrasmall superparamagnetic iron oxide for diagnostic imaging. Besides being a targeting agent, SM5-1 has been proved to be able to inhibit tumor cell growth or induce apoptosis [78].

### 6.2. Carbohydrate-Targeting Agents

Due to the prevalence of carbohydrate-binding receptors on hepatocytes, carbohydrate can also be used as targeting agents for HCC. Xue et al. delivered RASSF1A gene using a dual targeting approach through galactosylated-carboxymethyl chitosan-magnetic iron oxide NPs (Gal-CMCS-Fe_3_O_4_-NPs) [133]. The galactosylation targets the asialoglycoprotein receptors (ASGP-Rs), while an external magnetic field improves the efficiency of the gene therapy, potentially by directing the NPs to the target site [20]. ASGP-Rs are highly expressed on HCC cells. Xu et al. also used a galactose moiety in the form of galactosylated DOPE, which was conjugated using lactobionic acid, to target the HCC cells. The uptake into the cell was controlled by ligand-receptor binding, and endocytosis was mediated by clathrin. Liu et al. covalently conjugated a hydrophilic glucosyl-galactose disaccharide, lactose, to DOX in order to target the ASGPRs [65]. Wei et al. investigated Lactoferrin (Lf) to target HCC cells as Lf is able to bind to the ASGP-R as well [134,135]. Galactosamine is another target for the ASGP-R. Shen et al. conjugated this target to albumin NPs and used it to delivery DOX [93,111]. Similarly, Liang et al. delivered PTX using poly(*γ*-glutamic acid) and poly(lactide) NPs with galactosamine conjugation to target the liver cancer cells which resulted in specific interaction with the hepatoma tumor in mice via ligand-receptor binding. This targeted chemotherapy demonstrated enhanced reduction of the tumor in comparison to the non-targeted NP-PTX therapy [59]. Zhang et al. [57] targeted the ASGP-Rs as well. The pullulan polysaccharide backbone in their biomaterials bind naturally to ASGP-Rs as shown in Figure 5 [136]. Ni et al. used a dual targeting approach to target HCC cells [60]. Poly(ethylene glycol)-poly(lactic-co-glycolic acid)-poly(ethylene glycol) (BLPP) copolymer NPs were synthesized and conjugated with biotin-/lactobionic acid for targeted delivery of curcumin- and 5-fluorouracil. Due to the dual targeting, a higher cellular uptake was seen in HCC cells, and a lower residual accumulation was seen in normal tissue in comparison to the single targeting and non-targeting NPs [60]. Similarly, Abdelmoneem et al. used lactobionic acid and folic acid to target nanomicelles loaded with the phytochemicals berberine (BRB) and diosmin (DSN) to HCC cells [85].

### 6.3. Peptide/Protein-Targeting Agents

Monoclonal antibody have shown clinical potential, however its associated with impaired tumor penetration [82]. Therefore, peptide-targeting agents have been developed to overcome this. Lo et al. identified SP94, as a peptide targeting agent specific for HCC [82]. This peptide was conjugated to PEGylated liposome and used to deliver DOX. The peptide-targeting agent can decrease hematologic toxicities by avoiding the reduction in the total WBCs. When administered in vivo, there was significantly more SP94 accumulated at tumor sites than other organs. Bibby et al. delivered DOX with a NPs with a core made out of insulin multi-methacrylate with a targeting peptide, cyclic RGD [96].

### 6.4. Enhanced Permeability and Retention Effect (EPR)

Several biomaterials themselves often result in the selective accumulation of these vehicles and their payload into tumors instead of normal tissue due to the enhanced permeability and retention effect (EPR) [137]. CNPs are a prime example of this [67,68]. Particle size plays a role in the accumulation of particles in the liver. Smaller particles are more likely to be internalized and aggregate at the tumors due to the enhanced permeability and retention effect (EPR) [138]. However, larger NPs (>200 nm) are more likely to be recognized by monocytes and the reticuloendothelial system [139]. Particles made with gold also acts as its own targeting agent for HCC as gold accumulates in the liver [75]. Tomuleasa et al. [77], Paino et al. [74], and Ma et al. [78] did not use gold as only the bioactive factor, but also as the targeting agent. Instead of using the biomaterial as the targeting agent, Ma et al. also conjugated the gold NP with SM5-1, which targets an overexpressed surface protein, p30 in HCC [78]. Zhou et al. also demonstrated that their dendrimers NPs localize to liver site, not depending on a specific targeting agent [95]. However, the cell type-specific uptake and intracellular release depended on chemical structure of the different dendrimers.

## 7. Biomaterial-Based 3D In Vitro Models to Study Liver Cancer

### 7.1. 2D Versus 3D Cellular Models for HCC

**Figure 10:**
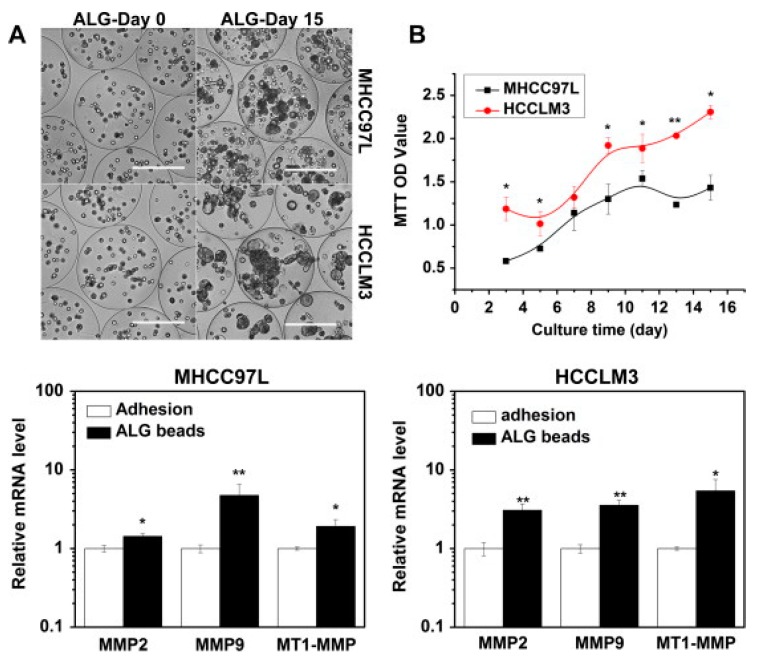
The growth profile and metastasis-related gene expression profile of HCC cells cultured in alginate beads. (**A**) The morphological appearance of MHCC97L and HCCLM3 cells, at day 0 and day 15. Scale bar: 200 μm. (**B**) Proliferation curves by MTT assay. Quantitative real-time PCR analysis graphs in the bottom side of the figure show gene expression of metalloproteinases (MMPs). β-Actin was used as an internal control. Reprinted with permission from Elsevier, Xu et al., *Exp. Cell Res*, 2013 [143].

In recent years, the increased development of biomimetic scaffolds for various biological applications has shown that the behavior of cells in three dimensional (3D) is very different from that of the same cells in bidimensional (2D) structures [140]. The remarkable effect played by the third dimension over cell growth has been observed in smaller and simpler cellular aggregates, such as spheroids, up to more architecturally complex biomaterial-based porous scaffolds [141]. Both the cellular genotype and phenotype, as well as the various biological phenomena (e.g., proliferation, differentiation, function), have been found to greatly differ in the two culture methods. These diversities can be even more evident when comparing the two models according to cancerous cells. As an example, several studies have shown that the 3D culture of tumor cells leads to a worsening of the malignant phenotype and an increase in drug resistance if compared to 2D culture, thus suggesting that 3D models reflect the phenomenology of in vivo tumors much more faithfully [142,143] (Figure 10).

From an historic perspective, the first studies related to the development of 3D in vitro models for liver aimed to create structures for tissue engineering and organ transplantation [144]. Subsequently, these models were also used for the evaluation of the efficacy and cytotoxicity of drugs, as well as for the study of cell metabolism and pathological phenomena. Initially, the cells grown on these 3D supports were primary hepatocytes from rat or, most rarely, isolated from a patient. Indeed, the difficulty in isolating human cells and the poor survival capacity of primary liver cells in culture have led researchers to use cell lines derived from hepatocellular carcinoma (HCC), such as HepG2, HepaRG, Huh7, and others [145]. The behavior of these lines appeared to be very similar to that of primary cells with respect to the morphological aspect (e.g., cellular polarity), the synthesis of molecules such as albumin and urea, the activity of detoxification enzymes, and the formation of similar structures in the biliary canaliculi. Moreover, the 3D culture of these cell lines in the form of spheroids has also been applied in researches aimed to obtain more detailed information on the molecular mechanisms of liver tumor development and for the evaluation of the pharmacological response to new anticancer drugs [144]. More recently, spheroids have evolved into organoids, which are obtained by inducing the aggregation of multiple cell types (e.g., endothelial, mesenchymal, hepatic) into macroscale (millimeter size) constructs acting as functional units [146]. Organoids have also been derived from liver cancers [147]. However, due to their compactness, these organoids are usually vital only for a limited timeframe in vitro. To have longer usable time points, scientists therefore use to disaggregate and reform the organoids over time, like cells in culture.

The great advantage of biomaterial-based scaffolds over spheroids and organoids is multifold: (1) allows for a better tissue biomimicry by providing a preliminary artificial extracellular matrix (ECM) on which the cells can grow and assemble in a natural fashion, (2) provides mechanical and architectural cues to which the cells can respond with a more reliable function, (3) can impart chemical signals thanks to the chosen biomaterial and/or biomolecules, (4) permits cell survival for long times thanks to its porosity, and (5) enables the generation of complex multicellular constructs in which diffusion gradients can be consistent to cell viability and function [142]. For the development of 3D culture models of HCC, both natural and synthetic materials have been used to obtain biodegradable and biocompatible scaffolds with a porosity suitable for cell proliferation and microvasculature formation and infiltration. The first scaffolds used in this research area have been produced starting from the (co)polymers approved by FDA for biomedical applications, namely, polyglycolic acid (PGA), PLLA, PLGA, PCL, and some others [148,149]. These biomaterials were processed into porous scaffolds prepared via different techniques, including rapid prototyping and solvent evaporation combined with particle elimination and cultured with hepatocytes for liver regeneration [150]. Only more than a decade later, the concept of tissue engineering, which is inherently based on the use of biomaterial-scaffolds, has been transferred to cancer research [151,152,153,154].

### 7.2. Hydrogel-Aided Spheroids for HCC

Spheroids are artefacts obtained in cell culture leading to compact cellular microspheres with a broad size range (20–1000 µm) which have become largely used in cancer research [142,155]. In order to improve the reliability of the results obtained with the spheroid model, many 3D cultures of liver tumor cells are availing themselves of hydrogels based on collagen, alginate or gelatin, containing molecules or growth factors immobilized in the structure via different chemical processes, even though also synthetic hydrogels are recently gaining attention for their peculiar properties, such as stimuli responsiveness [143,156,157,158,159,160,161]. Within these hydrogel structures, spheroidal masses or cell aggregates are formed under non-adherence conditions, in which the ECM-cell interactions are very narrow and grant for a better communication of intracellular transduction signals. Inherently, spheroids can only partially mimic the tumor microenvironment (TME) in terms of ECM secretion, interaction between cancer cells, diffusion gradients therefore increased resistance to treatments, whereas they lack of other TME features, including phenotype diversity and tissue architecture [155,162]. Moreover, difficulties in controlling spheroid size and obtaining consistent outcomes from assays have been reported, therefore many efforts are being reported on the standardization of spheroid size and function [163,164]. Spheroids thus represent a primary model of 3D cell culture, which for its simplicity can still contribute to cancer investigations and can give preliminary insight on 3D phenomena [155,162,163].

**Figure 11:**
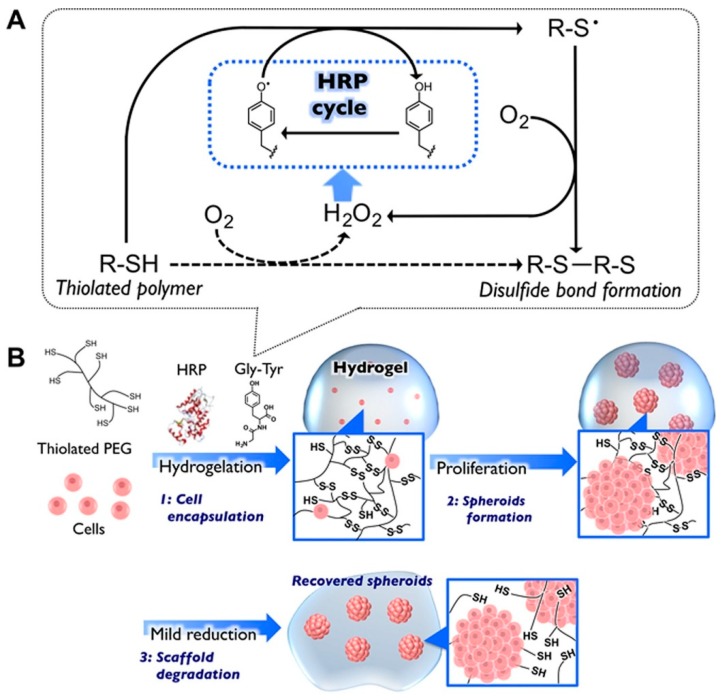
(**A**) Fabrication of a redox-degradable hydrogel by using horseradish peroxidase (HRP) catalysis: self-oxidation of a thiolated polymer generating hydrogen peroxide, hydrogelation (dashed arrows), HRP-mediated phenoxyradical formation promoting disulfide bond between the thiolated polymers (solid arrows). (**B**) Schematic of the fabrication and the recovery of cellular spheroids using redox-responsive hydrogels: encapsulation of target cells, spheroid formation by cell proliferation, recovery of the spheroids by degrading the scaffolds under reductive conditions. Reprinted with permission from Wiley, Moriyama et al., *Biotechnol. J.*, 2016 [159].

There are several works reporting on hepatic cells that better express their function in spheroid culture, and different synthetic hydrogels have been used to better control shape, size and formation of spheroids due to their stimuli-responsiveness to specific external triggers, therefore reacting to dynamic microenvironments [159,160]. For example, a redox responsive hydrogel based on PEG allowed increased liver-related functionalities, in terms of albumin and urea synthesis, in spheroids of the hepatoma cell line HepG2 [159]. A covalent disulfide hydrogel network based on a star-branched PEG derivative (8-arm PEG-SH) served for the formation of HepG2 cell spheroids. Cell recovery was also possible thanks to the polymer biodegradation under mild reducing conditions exerted by cysteine (Figure 11).

Another study has reported on the use of a temperature responsive hydrogel based on poly(*N*-isopropylacrylamide) (pNIPAAm)-*co*-gelatin for spheroid formation, in which the hepatoma cell line Hepa/8F5 derived from a genetically engineered mouse model showed higher function with respect to the 2D cell counterpart, according to albumin synthesis and other metabolic activities [160]. In addition, Hepa/8F5 cell spheroids grown in presence of pNIPAAm after doxycycline induction become drug-resistant, not only to tamoxifen but also to acetaminophen. This cytotoxicity study, among others, pointed out the protective role of 3D cell aggregation as a diffusion barrier to toxic molecules [155,160,164,165]. The tight cellular 3D aggregation in spheroids brings both positive and negative effects: on one hand, cells show improved secretive and metabolic functions; on the other hand, the diffusional limitations inhibit molecular trafficking, which in turn has consequences on the spheroid vitality [164].

The phenomena of cellular necrosis due to hypoxia in spheroids in some cases has been considered a limiting factor for the use of hydrogels. However, hypoxia-induced cell death has been found in the in vivo tumor environment in which the cells are deprived of oxygen and modify their behavior by developing a more aggressive phenotype [166]. In an instructive study, some researchers have developed a 3D model based on a collagen hydrogel. Co-cultures of HepG2 cells and stromal fibroblasts have been used to obtain hetero-spheroids, namely, spheroids formed by both tumor cells and stromal cells, of uniform and controlled size [161]. Subsequently, these were encapsulated in a collagen hydrogel to create a 3D model of HCC also used for the evaluation of the anti-tumor drug response to DOX. The results of this study have shown that the formation of hetero-spheroids is able to mimic some features of the in vivo TME, in which viable, proliferating, and functionally active cells, as demonstrated by cytochrome P450 activity, were observed.

DOX treatment showed higher chemoresistance in hetero-spheroids than in 2D culture conditions. This phenomenon seems to be related to the compactness of the spheroid structure, in which the cells layering on the outer surface would act as a barrier by hampering the penetration of the drug in the innermost layers, and to the hypoxic area at the central level of the spheroid, which would modulate the response of the cells by enhancing their endurance and vitality [155,160,161,164,165]. The increased resistance to the DOX action has also been attributed to the presence of collagen encapsulating the spheroids, as a further physical barrier to drug diffusion which at the same time better supports cell vitality and proliferation thanks to cell-ECM interactions [161,164].

**Figure 12:**
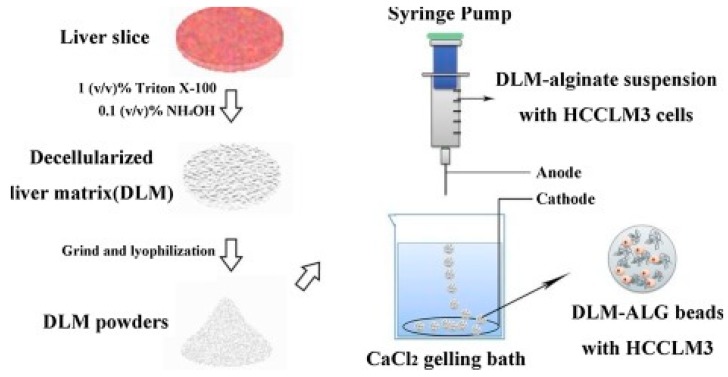
Schematic showing the preparation of decellularized liver matrix (DLM) and DLM-alginate hybrid gel beads (DLM–ALG beads). Reprinted with permission from Elsevier, Sun et al., *Int. J. Biol. Macromol.,* 2018 [170].

Xu and collaborators have used alginate hydrogels to evaluate in detail the possible cellular and molecular mechanisms that preside over the development of HCC metastases [143]. Alginate is a polysaccharide derived from brown algae which is widely used for its simplicity to obtain biocompatible hydrogels [167]. The structure of these gels is obtained by ionic crosslinking with bivalent cations, such as Ca^2+^, in order to control their mechanical properties and enable a better recovery of the spheroids [168]. In the study reported by Xu et al., two HCC cell lines, one with low metastatic potential (MHCC97L), the other with high infiltrating capacity (HCCLM3), were encapsulated in alginate spheres. The results highlighted that the cells grown in the spheroids reached an increased maturity in the 3D culture with respect to the monolayers. This increase was even more evident in the cells retaining high metastatic potential, thus suggesting that this alginate hydrogel is able to structurally and functionally mimic the hepatic TME [143] (Figure 10). Indeed, it has been largely reported that alginate is a valid biomaterial for long term hepatocyte cultures, including HepG2 cells [156,169]. Alginate can easily incorporate biological molecules to better mimic the liver TME (Figure 12). Sun et al. developed decellularized liver ECM-alginate hybrid gel beads for HCCLM3 cell culture by using a simple setup, demonstrating increased cell viability over time as a model of study for liver cancer metastasis [170]. Concerning the dimensional control of the spheroids, Lau and coworkers have indicated that alginate hydrogels can serve for entrapping gelatin microspheres so as to embed HepG2 cells in a large amount [157]. After encapsulation in the hydrogel, the genipin cross-linked microspheres were dissolved using metalloproteinase-9 (MMP-9) to create cavities with a predefined diameter, not exceeding 200 µm, in which the cells could form small-sized spheroid aggregates. In this way, the problem of formation of necrosis areas within the spheroidal masses due to the limits of nutrient diffusion was better overcome [157,164]. Being a polysaccharide, alginate can be easily mixed with other natural-origin hydro soluble biopolymers. Leung et al. have used alginate blended with chitosan, a natural polysaccharide derived from the partial deacetylation of chitin, known to share some structural similarities with the glycosaminoglycans present in the ECM [158].

The use of this biomaterial combination allowed a porous and interconnected 3D structure to be created, in which the characteristics of biocompatibility, biodegradation, non-immunogenicity, and ability to stimulate cell differentiation were contemporaneously achieved. In particular, this study aimed to carry out a pharmacological screening by cultivating two cell lines, HepG2 and PLC/PRF/5, both on the chitosan-alginate scaffolds and 2D Matrigel^®^, the latter being gelatinous protein mixture secreted by mouse sarcoma cells commonly used in cancer laboratory research [171]. The findings reported by these authors have highlighted the ability of this 3D models to stimulate the formation of cell aggregates with an increased expression of molecules related to tumor malignancy and ability to metastasize. The obtained data also in this case confirmed that the 3D models enhanced cell insusceptibility towards the chemotherapeutic drugs [158,161]. Furthermore, it was shown that the cell-hydrogel complex implanted in the animal was able to induce a strong vascularization [158].

All the above-mentioned studies reporting on HCC spheroids obtained using hydrogels documented the improved ability of these 3D structures in recapitulating functional and chemoresistance traits of HCC, which is poorly found if the same cells are cultured in 2D. From this point of view, spheroids represent an easy and valuable model for HCC drug screening [158,160,161,172]. Collectively, these findings also highlighted the key role played by biomaterials, synthetic, biological, and bioartificial hydrogels in facilitating spheroid formation, size control, and cell recovery, in addition to enhancing cell–cell and cell–matrix interactions, which in the end catalyze a more reliable cell function and drug susceptibility. In some cases, cell necrosis, frequently observable in spheroid models, can replicate pathological aspects and therefore be still useful in cancer research. However, the intrinsic nature of spheroids as artificially aggregated cell clusters is revealed by lack of morphological similarity with the native tumor, which in the end hampers more sophisticated observations to be taken from this 3D model.

### 7.3. Biomaterial-Based Scaffolds for HCC

The concept of cancer tissue engineering has only recently come to a diffuse scientific interest, but has attracted great attention [142,151,152,153,154]. Biomaterials-based supports endorsing the concept of tissue engineering, which relies on porous scaffolds acting as a preliminary synthetic ECM, can be exploited to better comprehend 3D cell behavior [173]. Conversely from spheroids, in these artificial microenvironments provided with porosity and topographical and mechanical cues, cells can find their own 3D organization and hierarchical structure. The scaffolds can be provided with different architectures by tissue-engineers—e.g., spongy or fibrous, smooth or nanopatterned—thus resembling specific traits of a tissue of interest [149,173]. Multiple cell populations can also be seeded on these structures, with clinically relevant dimensions (e.g., 5–8 mm size), to reach even complex macroscopic 3D models [174]. Starting from early regenerative goals in the eighties, in recent years tissue engineering has been evolving also towards in vitro tissue disease modeling, including cancer [142]. Only a few review papers on cancer tissue engineering can be found before 2010, thus confirming the novelty of this research field [152,153]. 3D scaffolds serve as an artificially created ECM able to provide a preliminary structure for cell organization and growth, which can be highly instructive for the study of cancer TME [142,151,153]. Indeed, both natural and synthetic origin scaffolds, provided with proper physico-chemical, architectural, and mechanical features can provide biomimetic cues for tumor cell growth [152,154]. Specifically, only a limited number of scaffold types have been reported, which can give a preliminary insight on the role played by different biomaterials, mechanical properties and structural architectures on HCC cell behavior, including, synthetic, biologic, composite (intended as multiphase) and bioartificial (intended as synthetic and biologic blends of copolymers) [172,175,176,177,178,179,180,181].

As a first example, in 2007, Bokhari and collaborators have developed a 3D porous scaffold based on polystyrene to culture HepG2 cells [172]. These authors employed a synthetic scaffold previously developed for osteoblast growth, fabricated via high internal phase emulsion (HIPE) polymerization, which is known as PolyHIPE Polymer (PHP). In this type of scaffold, in which the pore size and the porosity can be tuned by changing the chemical composition of the emulsion and the processing conditions, thus achieving maximum values of a few hundred microns and 97%, respectively [172,182]. The morphological evaluation of the HepG2/PHP constructs showed that the cells were viable, in the proliferative phase, and were organized in aggregates enabling the formation of bile ducts rarely observable in 2D cultures. Moreover, it was observed that the function of the cells grown on the porous matrix was increased if compared to the monolayer culture, whereas, the 3D culture was less susceptible to treatment with methotrexate, a hepatotoxic drug [172]. Many authors have pointed out the need to investigate more in-depth the TME and the interactions of tumor cells with TME, in order to clarify the molecular mechanisms underlying tumor proliferation and metastasis, with particular emphasis to liver cancer [183].

Using the sol–gel method and leachable sucrose particles, Kataoka et al. proposed an organic–inorganic scaffold constituted of tetraethoxysilane (TEOS) and polydimethylsiloxane (PDMS) [176]. The authors indeed used commercial PVA scaffolds (from Able Co., Tokyo, Japan) with pore size (130 µm and 200 µm) and porosity (about 90%) similar to those of their own scaffolds. In TEOS–PDMS scaffolds, the HepG2 cells showed enhanced proliferation and clustering capacity with respect to PVA scaffolds, which the authors attributed to the poral properties of PVA (e.g., smooth pore surface and reduced size of interpore openings), supposed to impair an efficient cell clustering in dynamic flow conditions as a consequence of the higher upstream static pressure under constant flow, if compared to TEOS–PDMS scaffolds. As a result of 3D cultures, albumin secretion in was about 3-fold higher than in 2D cultures and independent of the scaffold polymer [176].

Among natural origin scaffolds used for liver cancer, Kundu and collaborators have reported on silk fibers chosen for the mechanical characteristics of the material and the properties of fibroin, a biocompatible silk protein, to naturally express the amino acid residues Arg-Gly-Asp (RGD), as signaling molecules [177]. This amino acid sequence is present in the native extracellular collagen and the cells are naturally able to bind it by surface integrins, showing improved viability [184]. These scaffolds showed pores distributed in a range of 40–130 µm, values considered standard for nutrient diffusion and cell infiltration. A particular HCC cell line was cultivated on this 3D substrate, HepR21, which expresses at high levels the hyaluronic acid binding protein 1 (HABP1), a protein recognized to be involved in the progression of the tumor and in the regulation of the metabolism of the tumor cell [177]. The HepR21 cells also express high levels of hyaluronan, a component of ECM typical of TME, which enables the formation of an extracellular reticulum, thus promoting cancerous cell proliferation and metastasis [185]. The results of this study have proved that the HepR21 cells were capable of adapting to the 3D support, forming aggregates evenly distributed in the scaffold and showing a proliferative capacity and infiltration higher than the control cells (HepG2) cultivated under the same conditions. Furthermore, the culture of these cells morphologically and functionally reflected the long-term culture characteristics of solid tumors in vivo [177]. Silk fibroin was blended with chitosan, frozen and lyophilized to obtain silk fibroin/chitosan scaffolds apt for HepG2 cell growth [181]. In this system, the final porosity and mechanical properties of the scaffolds can be tuned by varying the blend composition, thus leading to scaffolds with porosity > 95% and pores with size within 100–150 µm, if the concentration of biopolymers in the mixture is < 6% *w*/*w*. Even if this study of She and colleagues was driven by a regenerative purpose, it was shown that HepG2 cells can intensively proliferate in these 3D matrices, thus suggesting novel uses for their application. In another study still aiming at liver tissue engineering, Gotoh and coworkers used silk fibroin conjugated with lactose to obtain Lac-CY-SF sponges able to target β-galactose residues, which represent hepatocyte-specific ligands [175]. These sponges displayed pores with variegated shape and size: round (100 μm) and elongated (250–450 long and 100–150 μm wide). The authors compared the function of FLC-4 cells cultured in Lac-CY-SF, plain silk fibroin and collagen sponges for 3 weeks. Interestingly, the transferrin and HNF-4α genes were only expressed by cells cultured in Lac-CY-SF sponges. These results highlight the specific responsiveness of cells to different biomaterial substrate and corroborate the potential of silk as an instructive biopolymer for HCC modeling for its capability to be blended and conjugated with other biomolecules [175,181]. In order to better mimic the liver tissue as a microenvironment for hepatic cell growth, including HCC cells, decellularized liver appears as an interesting scaffold. Mazza et al. were able to completely decellularize human liver with maintenance of the original parenchymal architecture [179]. The acellular tissue was cut into 125 mm^3^ cubic scaffolds which were seeded with hepatic stellate cells (LX2), hepatocellular carcinoma Sk-Hep-1 and HepG2. The cells, cultured for 3 weeks, were vital, proliferative, and able to remodel the scaffold ECM.

The analysis of HCC TME using a tissue-engineered model has also been performed by Liang et al., who developed a new strategy to control the rigidity of a type of collagen hydrogel scaffold, and minimize variations in gas permeability and the number of sites of accession [178]. In this study, a gel was created by crosslinking collagen fibrils with various concentrations of a copolymer based on PEG and succinic acid. The change in the mass ratio between the two polymers led to the formation of hydrogels with variable elastic compression modules (E_0_) in the range 0.7–4.0 kPa. HepG2 cells were embedded in these collagen-PEG supports and in pure collagen hydrogel by an on-site cross-linking reaction. The results obtained highlighted that the tumor cells were able to form cellular aggregates of different diameters and inversely proportional to the hydrogel rigidity. In fact, clusters with a diameter ranging in 30–330 µm were observed in the supports in which the E_0_ value was 0.7 kPa (corresponding to that of soft tissues such as fat), whereas clusters with a diameter within 20–60 µm were more represented in supports with an E_0_ value of 4 kPa (corresponding to that of healthy liver) [186]. The cells of the spheroids obtained in the soft hydrogel had a proliferative and infiltration potential much higher than those observed in the stiff hydrogel. In addition, the spheroids obtained in soft hydrogel showed an increase in β1-integrin expression, by resulting in increased cell adhesion to ECM, and VEGF, a factor responsible for the development of new vessels around the tumor [178]. The data obtained suggested that the rigidity of the scaffold was able to influence the phenotype and activity of the encapsulated cells [187,188].

**Figure 13:**
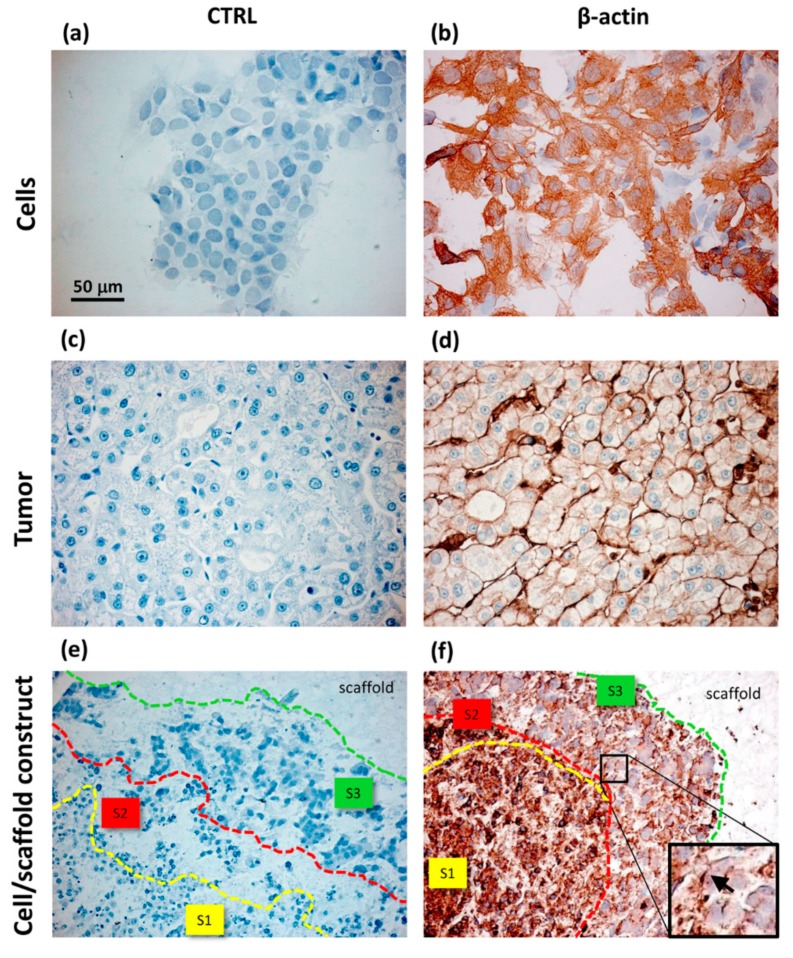
Immunohistochemical analysis of HepG2 cells cultured in monolayers (**a**,**b**); samples of HCC tumor (**c**,**d**) and HepG2 cells cultured inside PVA/G hydrogels (**e**,**f**). For each sample type, negative controls (**a**,**c**,**e**) and β-actin expression (**b**,**d**,**f**) are shown. S1, S2 and S3 in (**e**,**f**) define the areas of different morphotype localization within the cell/scaffold constructs. The insert in (**f**) shows a few cells with a lamellipodial-like expression of β-actin, indicated with an arrow.MDPI Creative Common Attribution license, Moscato et al., *J. Funct. Biomater.*, 2015 [180].

Among the hydrogels as 3D models of HCC have recently been studied are also those of PVA, a synthetic polymer capable of physical cross-linking at low temperatures (i.e., cryo-gelation) by forming lamellae that are consolidated through thermal cycles of freezing and thawing [180]. A suitable hydrogel was obtained by adding gelatin in the PVA solution to obtain a PVA/gelatin 80/20 (*w*/*w*) matrix with a final morphology that appeared similar to the liver parenchyma. By using bioartificial PVA cryogels, mixed with diverse biomolecules at different weight ratios, it is possible to slightly vary pore size distribution and scaffold inner morphology [189]. For PVA/gelatin blends, simple freeze-thawing allowed the largest pore volume to be occupied by pores with size lower than that obtained with emulsion and lyophilization [189,190,191]. Specifically, in the PVA/gelatin hydrogels prepared by Moscato et al., 71.59% ± 5.03% of the measured volume was due to pores with diameters ranging in 0.007–30 μm, while only 28.41% ± 4.57% of the measured volume derived from pores with diameters ranging in 30–150 μm [180]. Therefore, the pore size of these PVA/gelatin scaffolds greatly differ from that of the commercial pure PVA scaffolds used by Kataoka et al., which in addition is reported as non-distributed [176]. In PVA/gelatin hydrogels, proposed by the authors as scaffold models for the study of HCC cell migration, HepG2 cells were cultured up to 24 days. A morphological study highlighted the formation of macro-aggregates with cellular areas with different morphology. In particular, at the endpoint, together with a significant increase in cellular metabolism, a forward-front area was identified in which the cell-scaffold interactions appeared to promote the expression of α5β1-integrin and β-actin. The strong immunolocalization of these proteins on the lateral edge of the cell membrane placed on the forward-front, suggested the presence of lamellipodia apt for migration. The cells present in the central area of the aggregate, however, they were organized similarly to those found in HCC. The authors also reported an intermediate zone with signs of necrosis, unlike what happens in the spheroids where the necrotic area is usually the central one. This area could play a role in the secretion of factors that induce morphological changes in HepG2 cells in the forefront area (Figure 13).

As the lower pore size in this scaffold if compared to all the other ones reported in this section possibly reduced cell motility, a better observational window of migratory phenomena was made it possible, which could be useful for further investigations related to HCC invasiveness and progression. Table A2 summarizes the main findings per biomaterial, model (spheroid, bead, and tissue-engineered) and cell type (Appendix B).

The studies on biomaterials and scaffold types for HCC modeling are limited and still variegated. Up to now, the primary applications of tissue-engineered models rely on the generation of HCC or normal liver tissue, and no drug screening has been performed yet. Differently from spheroids, only in tissue-engineered constructs tissue-biomimetic cellular organization has been found [172,180]. Improved cell function can be generally observed in 3D culture models, both spheroids and tissue engineered constructs [159,160,161,175,176]. By applying biomaterial scaffolds as artificial ECMs, the instructive effects of substrate compositional, physical, architectural and mechanical factors has become evident, since in these substrates, HCC cells are able to take their own morphological and tumorigenic nature. As a remarkable example, HeG2 showed enhanced infiltrative behavior in soft scaffolds [178]. Tissue-engineered models offer complex and fascinating TMEs for HCC understanding and also more reliable therapeutic screening, which would offer intriguing opportunities to cancer researchers in the future.

## 8. Conclusion and Future Trends

Biomaterials approaches provide versatile and easily tunable properties that can be varied to treat and study HCC. The biomaterials span a wide range from nano to macroscale for systemic delivery, local delivery and mimicking tumor microenvironment. The application of biomaterials may be a promising method to repurpose existing HCC drugs that may have limited effect previously through systemic delivery. Future development of biomaterial-based implantable devices should focus on the local delivery of a combinational therapy instead of the delivery of one traditional method alone.

**Figure 14:**
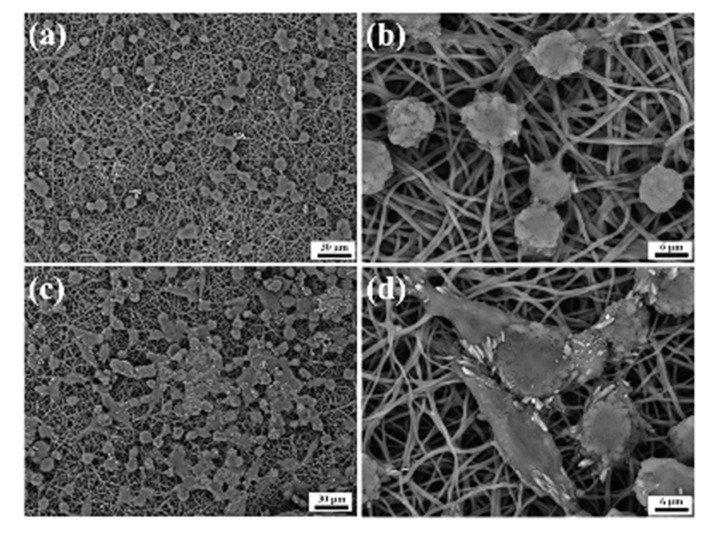
SEM images of HepG2 cells captured onto (**a**) mPEG-PVA/PEI-Ac and (**c**) LA-PEG-PVA/PEI-Ac nanofibers, respectively, after 240 min culture; (**b**,**d**) are high magnification image of (a,c), respectively. Reprinted with permission from Royal Society of Chemistry, Zhao et al., *RSC Advances*, 2015 [191].

Combining chemotherapy and anti-angiogenesis may result in synergistic ability of therapies to impeded tumor growth. Zhang et al. designed a stepwise pH-responsive NP system containing charge reversible pullulan-based (CAPL) shell and poly(β-amino ester) (PBAE)/PLGA core to deliver of PTX and combretastatin A4 (CA4) as a combination antiangiogenesis and chemotherapy to treat HCC [57]. Wang et al., studying methotrexate and combretastatin A4 in pH-sensitive pullulan-based NP carrier, enhanced antitumor and anti-angiogenic effects [91]. Future work can also focus on applying biomaterials to capture circulating tumor cells for cancer diagnosis applications. Zhao et al. used electrospun PVA/polyethyleneimine (PEI) nanofibers through a PEG mobilized with lactobionic acid onto for capturing hepatocellular carcinoma cells (Figure 14) [192]. 

Lactobionic acid can be used to capture ASGPR overexpressing HCC cells. In conclusion, biomaterial-based applications can deliver different bioactive factors systemically and locally and and/or serve as tissue engineering 3D models to better understand cancer biology and find more effective therapies for HCC. Recently, primary liver cancer-derived organoids are becoming tool for personalized biomarker identification and drug screening, owing to their ability to reflect the genetic complexity of the tumor [192]. It has been shown that hepatic and pancreatic cancer cells can grow in the form of organoids (also known as tumoroids) inside ECM-based gel as a natural biomaterial [193,194]. Combination of architectural cues (e.g., topography, porosity, mechanics) provided by engineered scaffolds and specific signaling molecules, such as those obtained from cell pre-generated ECM or lyophilized tissues, could be integrated to generate a 3D in vitro platform enabling better HCC understanding, thus maximizing the impact for and personalized as well as precision therapies.

## Dedication

“Dedicated to Maurizio Andruetto”.

## Appendix A

**Table A1:** Biomaterials that have been investigated for drug delivery for liver cancer.

Form	Biomaterial	Bioactive Factor	Targeting Agent	Type of Therapy	Source, Year
*Nanodiamonds*					
Nanodiamond	Carbon	Epirubicin		Chemotherapy	Wang et al., 2014 [53]
*PLGA particles*					
Nanoparticles (NPs)	Charge reversible pullulan-based (CAPL) shell and poly(β-amino ester) (PBAE)/poly(lactic-*co*-glycolic acid) (PLGA) core	Paclitaxel (PTX) and combretastatin A4 (CA4)	Polysaccharide pullulan backbone	Anti-angiogenesis and chemotherapy	Zhang et al., 2016 [57]
NPs	Poly d,l (lactide-coglycolide) (PLA)	5-fluorouracil (5-FU)	Anti-SM5-1	Chemotherapy	Ma et al., 2014 [56]
Microspheres	Poly(d,l-lactic-co-glycolic acid) (PLGA) core surrounded by a poly(L-lactic acid) (PLLA) shell layer	Doxorubicin (DOX) and/or chitosan-DNA NPs (chi-p53)		Chemotherapy and gene therapy	Xu et al., 2013 [58]
NPs	Poly(gamma-glutamic acid)-poly(lactide)	PTX	Galactosamine	Chemotherapy	Liang et al., 2006 [59]
NPs	biotin-/lactobionic acid modified poly(ethylene glycol)-PLGA-poly(ethylene glycol) (BLPP)	Curcumin (CUR) and 5-fluorouracil (5-FU)	Biotin/lactiobionic acid	Chemotherapy/Natural therapy	Ni et al., 2018 [60]
*Natural polymer-based particles*					
NPs	Gelatin	DOX-lactose	Lactose	Chemotherapy	Liu et al., 2018 [65]
Microspheres	Gelatin and chondroitin-6-sulfate	Interleukin-2		Immunotherapy	Hanes et al., 2001 [66]
NPs	Chitosan	None		Anticancer	Qi et al., 2007 [68]
NPs	Chitosan	None		Anticancer and anti-angiogenesis	Xu et al., 2010 [67]
NPs	Chitosan	Trans-resveratrol	Biotin and avidin	Phytochemicals	Bu et al., 2013 [69]
NPs	Chitosan	Plasmid DNA with granulocyte-macrophage colony stimulating factor, interleukin 21, internal ribosome entry site, and retinoic acid early transcription factor-1	Biotin	Gene therapy	Cheng et al., 2017 [70]
*Metallic particles*					
NPs	Galactosylated-carboxymethyl chitosan-magnetic iron oxide (Gal-CMCS-Fe3O4)	Ras Association Domain Family 1A (RASSF1A) gene	Galactose	Gene Delivery and Chemotherapy (Mitomycin injected as a free drug)	Xue et al., 2016 [72,133]
NPs	Ultrasmall superparamagnetic iron oxide	SM5-1	Anti-SM51	Immunotherapy	Kou et al., 2008 [73]
NPs	Gold	miR-375	Gold	Gene therapy	Xue et al., 2016 [75]
NPs	Gold conjugated with sodium citrate or polyamidoamine dendrimers (PAMAM)	None	Gold	Chemotherapy	Paino et al., 2012 [74]
NPs	Gold	SM5-1	Gold and selective binding of SM5-1	Immunotherapy	Ma et al., 2016 [78]
NPs	Gold with a monolayer of L-aspartate	DOX, cisplatin, capecitabine	Gold	Chemotherapy	Tomuleasa et al., 2012 [77]
*Lipid-based particles*					
Liposomes	Soybean phosphatidylcholine/cholesterol, (PEG)ylated	DOX	Lactoferrin	Chemotherapy	Wei et al., 2015 [79]
Liposome	PEGylated liposome (liposome material unclear)	DOX	Targeting peptide SP94	Chemotherapy	Lo et al., 2008 [82]
Immuno-liposomes	1,2-dioleoyl-3-trimethylammonium-propane (chloride salt) (DOTAP), cholesterol, 1,2-distearoyl-*sn*-glycero-3-phosphoethanolamine-*N*-[maleimide (polyethylene glycol)-2000] (DSPE-PEG-Mal),	Adriamycin (ADR) and ribonucleotide reductase M2 (RRM2) siRNA	Anti-EGFR Fab	Chemotherapy and Gene Therapy	Gao et al., 2013 [127]
Immuno-liposomes	1,2-distearoyl-sn-glycero-3-phosphoethanolamine-*N*-[maleimide(polyethylene glycol)-2000] (DSPE-PEG-MAL)	DOX	Anti-CD147 antibody (Metuximab)	Chemotherapy	Wang et al., 2018 [83]
Nanomicelles	Casein	Berberine (BRB) and Diosmin (DSN)	lactobionic acid (LA) and folic acid (FA)	Phytochemicals	Abdelmoneem, 2018 [85]
Liposomal NPs	Distearoylphosphatidylcholine (DSPC), cholesterol, dioleoylphosphatidylethanolamine (DOPE), distearoylphosphatidylethanolamine (DSPE)-mPEG2000, and DSPE-cyclic RGDfK	DOX or triple fusion gene for molecular imaging	Anti-CD44 antibody	Chemotherapy	Wang et al., 2012 [84]
Lipid NPs	Cationic lipid RL01, 1,2-Distearoyl-*sn*-glycero-3-phosphatidylcholine (DSPC), and 1,2-dimyristoyl-sn-glycero-3-phosphoethanolamine-*N*-[methoxy(polyethylene glycol)-2000] (DMPE-PEG2000) and cholesterol	Anti-miR-17		Gene therapy	Huang et al., 2017 [130]
Lipid NPs	Trimyristin (TM), egg yolk phosphatidylcholine (ePC), galactosylated dioleoylphosphatidyl ethanolamine (Gal-DOPE)	Docetaxel	Galactose and lactobionic acid	Chemotherapy	Xu et al., 2009 [87]
Lipidoid	Ionizable lipid or cationic lipid, disteroylphosphatidyl choline, cholesterol, and 1,2-dimyristoyl-*sn*-glycerol, methoxypolyethylene glycol	siRNA for all integrin subunits in hepatocytes		Gene delivery	Bogorad et al., 2014 [86]
*Other Particles*					
NPs	Hydroxyapatite	Selenium		Anticancer	Wang et al., 2016 [88]
NPs	Albumin	DOX	Galactosamine	Chemotherapy	Shen et al., 2011 [93]
NPs	*N*-urocanyl pullulan	Methotrexate and Combretastatin A4	Pullulan	Anti-angiogenic and chemotherapy	Wang et al., 2013 [91]
NPs	Polyisohexylcyanoacrylate (PIHCA)	DOX		Chemotherapy	Barraud et al., 2005 [94]
Dendrimer NPs	Lipids cholesterol, 1,2-distearoyl-sn-glycero-3-phosphocholine (DSPC), and lipid PEG2000 with amine core and thiol peripeheries	Let-7g microRNA		Gene delivery	Zhou et al., 2016 [95]
NPs	Calcium carbonate with lipid coating	Sorafenib (Sor) and miR-375		Chemotherapy and gene therapy	Zhao et al., 2018 [92]
NPs	Insulin multi-methacrylate	DOX	Targeting peptide, Cyclic RGD	Chemotherapy	Bibby et al., 2005 [96]
NPs	Block copolymer PEG5k-PLA8k	SN38 prodrug		Chemotherapy	Wang et al., 2018 [61]
*Local Delivery*					
Nanofibers NPs (immobilized on the nanofibers)	Poly(ε-caprolactone) disulfide cross linked branched PEI (ssPEI)	PTX and miRNA-145		Chemotherapy and gene delivery	Che et al., 2015 [98]
Microspheres	Glass	Phosphorus-32		Radiotherapy	Wang et al., 2008 [97]
Polymer millirods	Poly(lactic-co-glycolic acid) PLGA	DOX carboplatin 5-fluorouracil		Chemotherapy	Qian et al., 2002 & 2004 [101,103] Szymanski-Exner et al., 2003 [102]; Weinberg et al., 2007 & 2007 [99,100]
Drug eluting beads	Sulfonate-modified poly(vinyl alcohol) hydrogel	DOX sor		TACE, chemotherapy	Pawlik et al., 2011 [109]
Drug-eluting microspheres/beads (DEB)	Sulfonate-modified poly(vinyl alcohol) hydrogel	DOX		TACE, chemotherapy	Hong et al., 2006 [108]
Drug eluting beads	Polyvinyl alcohol polymer modified with sulfonate groups to form a hyodrogel	DOX		TACE, chemotherapy	Poon et al., 2007 [111]
Microspheres	Poly-lactide-co-glycolide PLGA	Mitomycin		TACE, chemotherapy	Qian et al., 2003 [110]
*Biomaterials to capture cells*					
Nanofibers	Lactobionic acid-functionalized electrospun polyvinyl alcohol/polyethyleneimine via PEG spacer	Diagnostic purposes		Cancer diagnosis	Zhao et al., 2015 [191]

## Appendix B

**Table A2:** Biomaterials that have been investigated for 3D models for studying liver cancer.

Biomaterial	Type	Model	Cells	Application	Results	Source, Year
Synthetic						
	PEG (8-arm PEG-SH)	Spheroid	HepG2	Spheroid formation and recovery due to polymer biodegradation with cysteine	Increased cell function (albumin and urea)	Moriyama et al., 2016 [159]
	Polystyrene (PHP)	Tissue Engineered (TE)	HepG2	Structure, function and cytotoxicity study (methotrexate)	TME bio-mimicry: bile duct formation and higher drug resistance	Bokhari et al., 2007 [172]
	TEOS–PDMS	TE	HepG2	Function under dynamic flow	TME bio-mimicry: increased proliferation and aggregation capacity, higher albumin synthesis than in 2D cultures	Kataoka et al., 2005 [176]
	PVA	TE	HepG2	Function under dynamic flow	TME bio-mimicry: higher albumin synthesis than in 2D cultures	Kataoka et al., 2005 [176]
Biologic						
	Collagen	Hetero-spheroid	HepG2 and stromal fibroblasts	Cytotoxicity study (DOX)	TME bio-mimicry: cell function (P450 activity) and increased drug resistance	Yip et al., 2013 [161]
	Alginate	Spheroid	MHCC97L HCCLM3	Metastatic mechanism study	TME bio-mimicry: increased cell maturity, in particular in metastatic cells MHCC97L	Xu et al., 2013 [143]
	Alginate/chitosan blend	Spheroid	PLC/PRF/5 HepG2	Cytotoxicity study (DOX)	TME bio-mimicry: increased malignancy and drug resistance	Leung et al., 2010 [158]
	Silk fibroin	TE	HepR21 HepG2	TME study	HepR21 showed irregular aggregation and higher proliferation capacity than HepG2	Kundu et al., 2013 [177]
	Silk fibroin/chitosan	TE	HepG2	Liver regeneration	Mechanical properties controllable, good cell proliferation	She et al., 2008 [181]
	Silk fibroin-lactose (Lac-CY-SF)	TE	FALC-4	Liver regeneration	Functional gene expression not found using collagen	Gotoh et al., 2011 [175]
Composite						
	Decellularized human liver tissue	TE	LX2 Sk-Hep-1 HepG2	Liver regeneration	Bio-compatibility and ECM remodeling	Mazza et al., 2015 [179]
	Alginate/decellularized liver ECM	Bead	HCCLM3	Metastatic mechanism study	TME bio-mimicry: Increased cell viability and metastatic potential due to liver ECM	Sun et al., 2018 [170]
	Alginate/gelatin microspheres	Spheroid	HepG2	Formation of spheroids with defined size by microsphere dissolution with MMP-9	Spheroids of 200 µm with no necrotic core	Lau et al., 2012 [157]
Bioartificial						
	pNIPAAm-co-gelatin	Spheroid	Hepa/8F5	Cytotoxicity study (tamoxifen and acetaminophen)	Increased cell function (albumin CYP3A4 activity, ammonia removal) and drug resistance	Sarkar et al., 2017 [160]
	Collagen-PEG/succinic acid	TE	HepG2	Effect of material stiffness	Higher cell cluster size and infiltration capacity in softer hydrogels	Liang et al., 2011 [178]
	PVA/gelatin	TE	HepG2	Cell migration	TME bio-mimicry: tissue-like cell organization, possibility to visualize migratory phenomena	Moscato et al., 2015 [180]
